# Integrated transcriptomic and metabolomic identification of the core pathway of photosynthetic carbon fixation in mung bean under saline-alkali stress

**DOI:** 10.3389/fpls.2026.1823453

**Published:** 2026-05-29

**Authors:** Kaibo Zhu, Xiaolei Li, Ruiwen Xue, Ruiqin Gao, Wenting Zhang, Hongchun Bao, Hailong Zong

**Affiliations:** 1 College of Agriculture, Inner Mongolia Agricultural University, Hohhot, China; 2 Agricultural Technology Promotion Center of Inner Mongolia Autonomous Region, Hohhot, China

**Keywords:** carbon fixation, metabolomics, mungbean, saline-alkali stress, transcriptomics

## Abstract

Mungbean (*Vigna radiata* L.), an important grain legume crop, suffers severe constraints in growth and yield under saline-alkali stress. However, research on the molecular mechanisms underlying stress responses remains limited, and the identification of key genes conferring saline-alkali tolerance is still in its exploratory stages. In this study, saline-alkali-tolerant material(JG)and saline-alkali-sensitive material(TQ)were subjected to 100 mmol·L^-^¹ mixed saline-alkali stress, with samples collected at 0, 3, and 7 treatment stages for analysis, and it was found that there were significant genotypic differences in superoxide dismutase activity and proline content after the treatment, while obvious differences in malondialdehyde content and soluble sugar content were observed in the same genotype before and after the treatment. Transcriptomic results revealed that the number of differentially expressed genes (DEGs) in each comparison group ranged from 1,833 to 7,886. A total of 42 differentially expressed transcription factors associated with saline-alkali stress responses were identified, and these transcription factors exhibited more pronounced stress-responsive expression patterns in the JG genotype. Weighted gene co-expression network analysis (WGCNA) was performed on the DEGs, which led to the screening of four core modules. The hub genes were further validated via quantitative real-time polymerase chain reaction (qRT-PCR), and the validation results were consistent with those of the transcriptomic sequencing. Notably, the hub genes involved in pyruvate metabolism and glyoxylate and dicarboxylate metabolism were upregulated with the prolongation of stress duration. Metabolomic analysis detected a total of 5,905 metabolites, and KEGG pathway annotation showed that the arginine and proline metabolism pathway was significantly enriched in all comparison groups. D-Arginine, D-Ornithine, L-Asparagine, spermine and nicotinamide riboside were identified as the key metabolites of mungbean in response to saline-alkali stress. Transcriptomic and metabolomic datasets were co-enriched in the carbon fixation in photosynthetic organisms pathway, where 18 DEGs were found to have regulatory or interactive relationships with key metabolites. Among these genes, *LOC106754509* and *LOC106772717*, which encode fructose-1,6-bisphosphatase, were both downregulated in the two genotypes after stress treatment, while *LOC106754931*, encoding aspartate aminotransferase, was upregulated in both genotypes. In addition, pyruvate content showed an opposite changing trend in the two genotypes. These findings provide important genetic resources and metabolomic data for an in-depth elucidation of the molecular regulatory mechanisms underlying saline-alkali tolerance in mungbean, clarify the key pathways of synergistic regulation between the transcriptome and metabolome, and lay a solid theoretical foundation for subsequent genetic improvement and molecular breeding of saline-alkali-tolerant mungbean.

## Graphical Abstract

**Figure:**
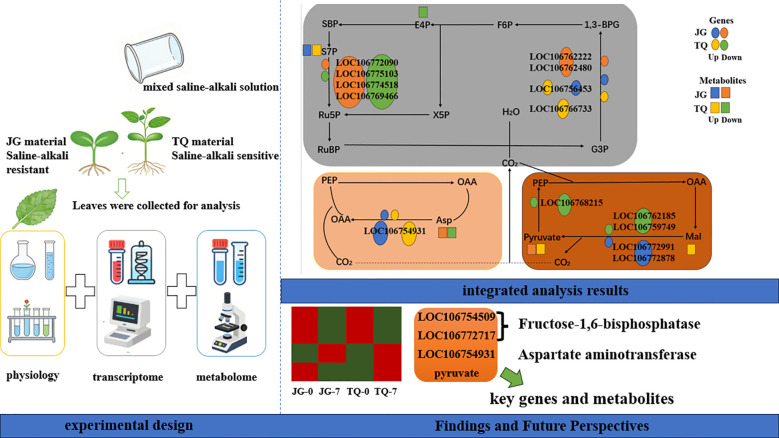
The abstract should ideally be structured according to the IMRaD format (Introduction, Methods, Results and Discussion). Provide a structured abstract if possible. If your article has been copyedited by us, please provide the updated abstract based on this version.

## 1 Introduction

Globally, over 1 billion hectares of land are affected by salinization, accounting for approximately 10% of total land area and distributed across more than 100 countries and regions, severely limiting crop distribution and productivity (Liu et al., 2026). Due to inappropriate farming practices and industrial pollution, the area of saline-alkali soil continues to expand (Huang et al., 2026). Saline-alkali stress is one of the major abiotic stresses limiting crop growth. Salinization leads to soil salt ion accumulation, causing soil compaction and hardening, which affects soil permeability and dramatically reduces water content, resulting in crop water deficit. Additionally, elevated root zone pH disrupts the proton gradient across membranes, affecting nutrient uptake and ion transport processes, ultimately leading to physiological metabolic disorders, restricted plant growth, or even death (Rengasamy, 2006; Flowers et al., 2015). To cope with saline-alkali stress, plants have evolved multiple strategies, such as producing organic osmolytes and excluding excess Na^+^ and Cl^-^ ions to alleviate saline-alkali-induced damage (Horie et al., 2012; Li et al., 2026). Mungbean (*Vigna radiata* L.) is an annual crop belonging to the family Leguminosae, subfamily Papilionaceae, tribe Phaseoleae, genus *Vigna*, with a long history of cultivation and consumption in China (Zahra et al., 2025). As a crop rich in high-quality dietary fiber, protein, vitamins, minerals, and polyphenols with both nutritional and medicinal values, mungbean is one of China’s important traditional export agricultural products (Ge et al., 2024). In recent years, with improved living standards and lifestyle changes, demand for minor grain crops has increased (Liang et al., 2025). Utilizing saline-alkali land for mungbean cultivation not only avoids competition with major crops for arable land but also effectively ensures the sustainable development of the mungbean industry.

With the advancement of high-throughput sequencing and its application in modern biology, numerous genes regulating abiotic stress responses in plants have been identified. Researchers have conducted in-depth transcriptomic analyzes of crops such as wheat (Cui et al., 2022), sorghum (Song et al., 2025), sugar beet (Li M et al., 2023), oat (Wang X et al., 2025), and rice (Jakada et al., 2025) under saline-alkali stress, identifying stress-responsive genes. To elucidate the molecular mechanisms of rice root response to salt stress, Jarvis et al., 2017 found that in the salt-tolerant variety Mulai, transporters participated in salt tolerance regulation after salt stress, while in the salt-sensitive variety IR29, transcription factors NAC, WRKY, and MYB were involved in transport regulation. Maughan et al., 2012 reported changes in transcript abundance of the ion transporter CqSOS1 in quinoa under NaCl solution stress. Metabolomics intuitively reflects overall changes in plants by studying metabolites (Zhang S et al., 2026) Currently, metabolomics has achieved significant research advances in abiotic stress studies of various crops including maize (Wang C. et al., 2025), wheat (Večeřová et al., 2022), sorghum (Ma et al, 2020), quinoa (Wang C. et al., 2025), and cotton (Li et al., 2015). Chen et al., 2021 studied the metabolome of rice seedlings under salt stress and identified 90 significantly changed substances, with L-asparagine serving as a key indicator for evaluating rice salt tolerance. Transcriptomics and metabolomics can explore biological questions from both gene expression and metabolic levels, mining key differential genes and differential metabolites in biosynthetic pathways. Han et al., 2023 integrated transcriptomics and metabolomics to discover that continuously upregulated differential genes in cotton after salt stress were mainly enriched in metabolic pathways such as flavonoid biosynthesis and amino acid biosynthesis, and the accumulation of cysteine, ABA, isopentenyl adenine-7-N-glucoside, and trehalose was crucial for cotton salt tolerance mechanisms, providing key biomarkers for salt stress tolerance.

To date, studies on saline-alkali stress in mung bean have mainly focused on the screening of saline-alkali-tolerant germplasm at the germination and seedling stages. Research on saline-alkali-tolerant genes remains relatively scarce, and no metabolomic studies have been reported. Breria et al., 2020 found that the proteins encoded by *Vradi07g01630* and *Vradi09g09510* may be functionally related to salt stress tolerance. The core metabolites and genes associated with saline-alkali stress in mung bean have not yet been reported. In the present study, transcriptomic analysis was employed to elucidate the expression characteristics of key transcription factors in mungbean under saline-alkali stress and to identify the core regulatory pathways involved. Metabolomic analysis was conducted to characterize the key metabolites responsive to saline-alkali stress and their accumulation patterns, as well as to screen out the core metabolic pathways. Furthermore, an integrated transcriptomic and metabolomic analysis was performed to investigate the mutual regulatory relationships between differentially expressed genes and differential metabolites. The findings of this study lay a solid theoretical foundation for the systematic elucidation of the molecular mechanisms governing mungbean’s response to saline-alkali stress.

## 2 Materials and methods

### 2.1 Experimental materials

The mungbean germplasm resources used were relatively saline-alkali-tolerant JG111-6 (JG) and relatively saline-alkali-sensitive Tuquan mungbean (TQ), obtained from preliminary laboratory screening and provided by our laboratory. Detailed measurement indices and screening criteria are provided in Tables A1, A2, **Supplementary Figure S9**.

### 2.2 Material cultivation

The experiment was conducted at the College of Agronomy, Inner Mongolia Agricultural University (45°5′N, 110°E). One hundred full, uniformly sized, pest-free seeds were selected from each material. Seeds were surface-sterilized with 30% H_2_O_2_ for 10 min and rinsed three times with distilled water. Purchased nutrient soil (neutral pH) was employed as the culture substrate. Each material was sown in 50 pots with 2 seeds per pot. Seeds were covered with 2–3 cm of substrate, leveled, and lightly pressed. Pots were placed in an artificial climate chamber under the following conditions: light intensity 400 μmol·m^-^²·s^-^¹, photoperiod (14/10) h (day/night), temperature (23/20)°C (day/night), relative humidity 50%-60%.

### 2.3 Experimental treatment

After the first trifoliate leaf of mungbean seedlings fully expanded, leaves from plants with consistent growth status and position were sampled, with 15 pots selected from each material, designated as stress period 0. Plants were subjected to stress treatment using a mixed saline-alkali solution at a concentration of 100 mmol·L^-^¹, in which the molar ratio of NaCl: Na_2_CO_3_: Na_2_SO_4_: NaHCO_3_ was 1:1:9:9, and the solution pH was 9.2. This ratio and concentration effectively simulate the conditions of soda saline-alkali soils in Inner Mongolia (pH 8.5–9.0). Applied by irrigation every 2 days with 50 mL each time. Leaves were collected after the 3rd and 7th stress treatments, designated as stress periods 3 and 7, respectively. Collected leaf samples were flash-frozen in liquid nitrogen and stored at -80 °C for later use. Soil pH and electrical conductivity at sampling are presented in **Table 1**.

**Table 1:** Soil pH and electrical conductivity (EC).

Materials/indicator	PH			EC (MS/cm)		
stage	0	3	7	0	3	7
JG	7.15	8.79	9.12	0.14	0.35	0.49
TQ	7.13	8.85	9.2	0.14	0.36	0.5

### 2.4 Physiological index determination

Leaves from all samples at three time periods were collected for physiological and biochemical index determination. Malondialdehyde content, proline content, soluble sugar content, and superoxide dismutase activity were measured using kits from Suzhou Grace Biotechnology Co., Ltd. Physiological data were statistically analyzed using SPSS 21.0 software. Significant differences among different treatments were tested by Duncan’s new multiple range test, with *P* < 0.05 indicating statistical significance.

### 2.5 Transcriptome sequencing and analysis

Collected leaves were frozen in liquid nitrogen and sent to OE Biotech Co., Ltd. for total RNA extraction, quality testing, cDNA library construction, and high-throughput sequencing with three biological replicates. After RNA sample quality verification, mRNA enrichment and cDNA library construction were performed, with library quality checked by Qubit 2.0, Agilent 2100, and q-PCR. Qualified libraries were sequenced on the BGI DNBSEQ-T7RS platform with PE150 read length. Raw image data files from high-throughput sequencing were converted to raw sequencing sequences (Raw Data) through base calling analysis, then filtered to obtain high-quality Clean data. HISAT2 software was used to align Clean Data with the mungbean reference genome (https://ftp.ncbi.nlm.nih.gov/genomes/all/GCF/000/741/045/GCF_000741045.1_Vradiata_ver6/GCF_000741045.1_Vradiata_ver6_genomic.fna.gz). StringTie was used for transcript assembly, with FPKM (Fragments Per Kilobase of transcript per Million fragments mapped) measuring transcript or gene expression levels. Gene functional annotation was performed using NR, KEGG, COG, KOG, GO, and other databases. Differentially expressed genes (DEGs) were screened using the DESeq2 software. To control the false positive rate (Type I error) resulting from multiple comparisons, the Benjamini-Hochberg method was used to adjust the raw *P*-values. Genes with Fold Change ≥ 2 and FDR < 0.05 were set as the screening threshold. Finally, GO enrichment analysis and KEGG metabolic pathway analysis were conducted on the screened differentially expressed genes. Volcano plots, bubble plots, and gene expression heatmaps were generated using the Majorbio cloud platform. Weighted gene co-expression network analysis (WGCNA) was performed on all DEGs from both materials. For WGCNA analysis, a soft threshold power of β = 30 was chosen. At this threshold, the signed R2 of the co-expression network was approximately 0.78, close to the recommended criterion of 0.8 in the WGCNA pipeline. Meanwhile, the average connectivity remained within a reasonable range, ensuring that the network satisfied the scale-free topology requirement. (**Supplementary Figure S1**).

### 2.6 Quantitative real-time PCR validation

To verify transcriptome data accuracy, qRT-PCR was used to validate the expression of 9 genes related to material saline-alkali resistance. Primers were designed via the NCBI website with length set at 18–25 bp, Tm value range of 58-62 °C, and product size of 80–200 bp. The reference gene Actin3 was used for normalization. Relative expression of target genes was calculated using the 2^(-ΔΔCt) method, with three biological replicates for each gene. SPSS 21.00 software was used for significance analysis, and Origin software for data visualization.

### 2.7 Metabolome sequencing and analysis

Leaves collected at three time periods were frozen in liquid nitrogen and sent to OE Biotech Co., Ltd. A pooled quality control (QC) sample was prepared by mixing equal volumes of all samples. Technical repeatability was evaluated by calculating the relative standard deviation (RSD) of peak areas for all metabolites in the QC sample. Detection was performed using ultra-high-performance liquid chromatography-tandem mass spectrometry (LC-MS/MS) for data acquisition. Mass spectrometry data were identified using public databases such as METLIN, HMDB, and OE’s proprietary database for qualitative and quantitative analysis. Based on OPLS-DA model parameter variable influence on projection (VIP value), Model evaluation parameters (R²Y and Q²) were reported simultaneously, and 200 permutation tests were additionally conducted to validate the robustness of the model. differential metabolites with VIP ≥ 1 and fold change (FC) ≥ 2 were screened. KEGG enrichment analysis was performed on screened differential metabolites.

## 3 Results and analysis

### 3.1 Physiological response of mungbean materials under saline-alkali stress

**Figure 1:**
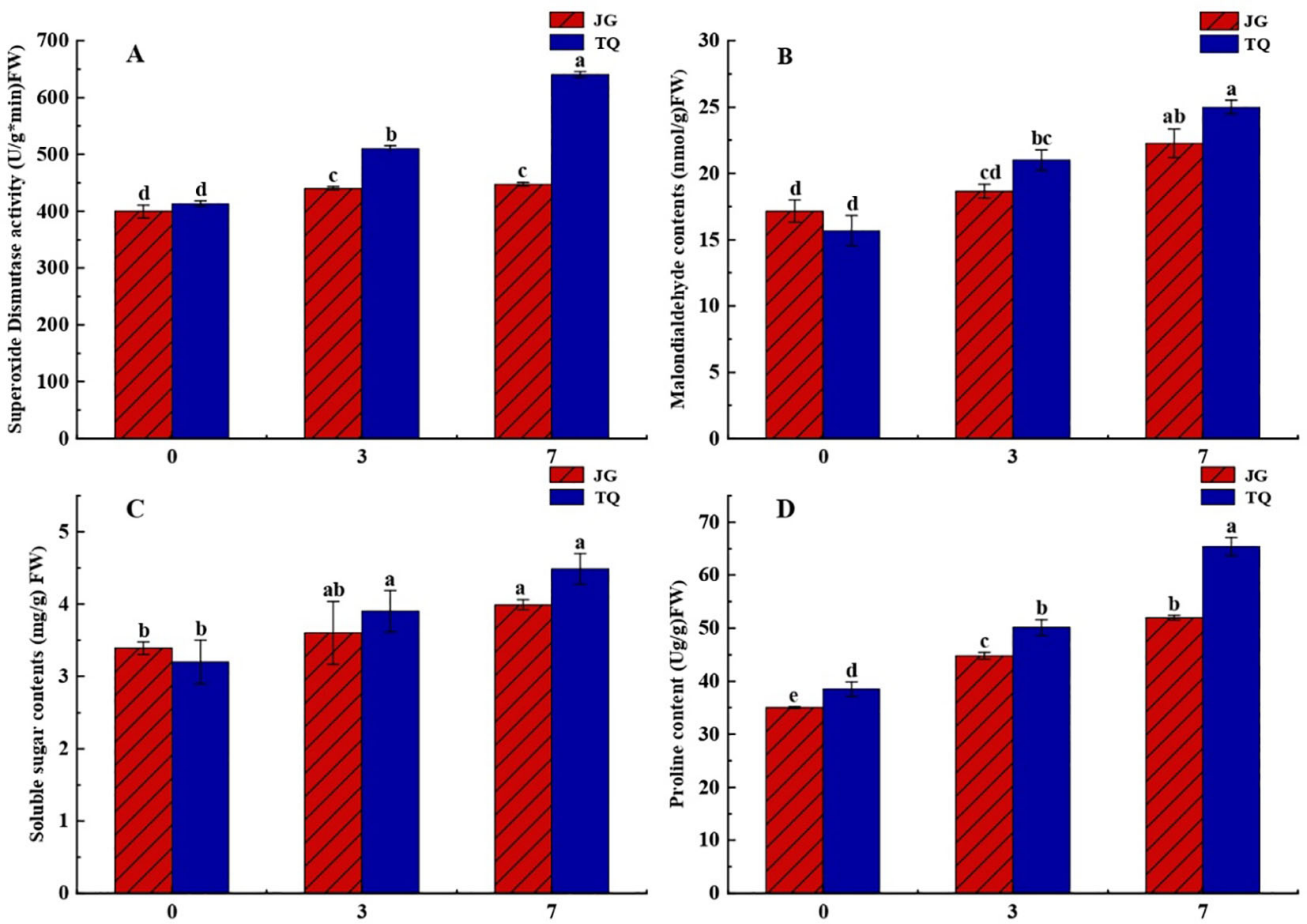
Physiological responses of mung bean (*Vigna radiata* L.) germplasm under salt-alkali stress. **(A)** Superoxide Dismutase activity (U/g*min) FW). **(B)** Malondialdehyde contents (nmol/g) FW). **(C)** Soluble sugar contents (mg/g FW). **(D)** Proline content (ug/g) FW). Different letters in the figure indicate significant differences (*P* < 0.05).

To investigate the responses of physiological indices of mung bean to saline-alkali stress, the leaf physiological indices were determined at the 0, 3 and 7 stages of stress treatment. For superoxide dismutase (SOD) activity, significant differences were observed between the 0 stage and the 3 and 7 stages in the JG genotype; SOD activity in the TQ genotype increased significantly with the extension of stress treatment time, and there were significant differences in SOD activity between the two genotypes at both the 3 and 7 stages (**Figure 1A**). With regard to malondialdehyde (MDA) content, a significant difference was found between the 0 and 7 stages in the JG genotype, while significant differences in MDA content were detected among all the three stages in the TQ genotype, and a significant genotypic difference in MDA content was observed at the 7 day stage (**Figure 1B**). For soluble sugar content, no significant differences were found between the two genotypes at the same stress stage, whereas significant differences were observed between the 7 and 0 stages in both genotypes (**Figure 1C**). Proline content increased with the prolongation of stress treatment time; significant differences in proline content were identified among different stages for the same genotype, and significant genotypic differences in proline content were also found at the same stress stage (**Figure 1D**).

### 3.2 Transcriptome changes in mungbean materials under saline-alkali stress

**Figure 2:**
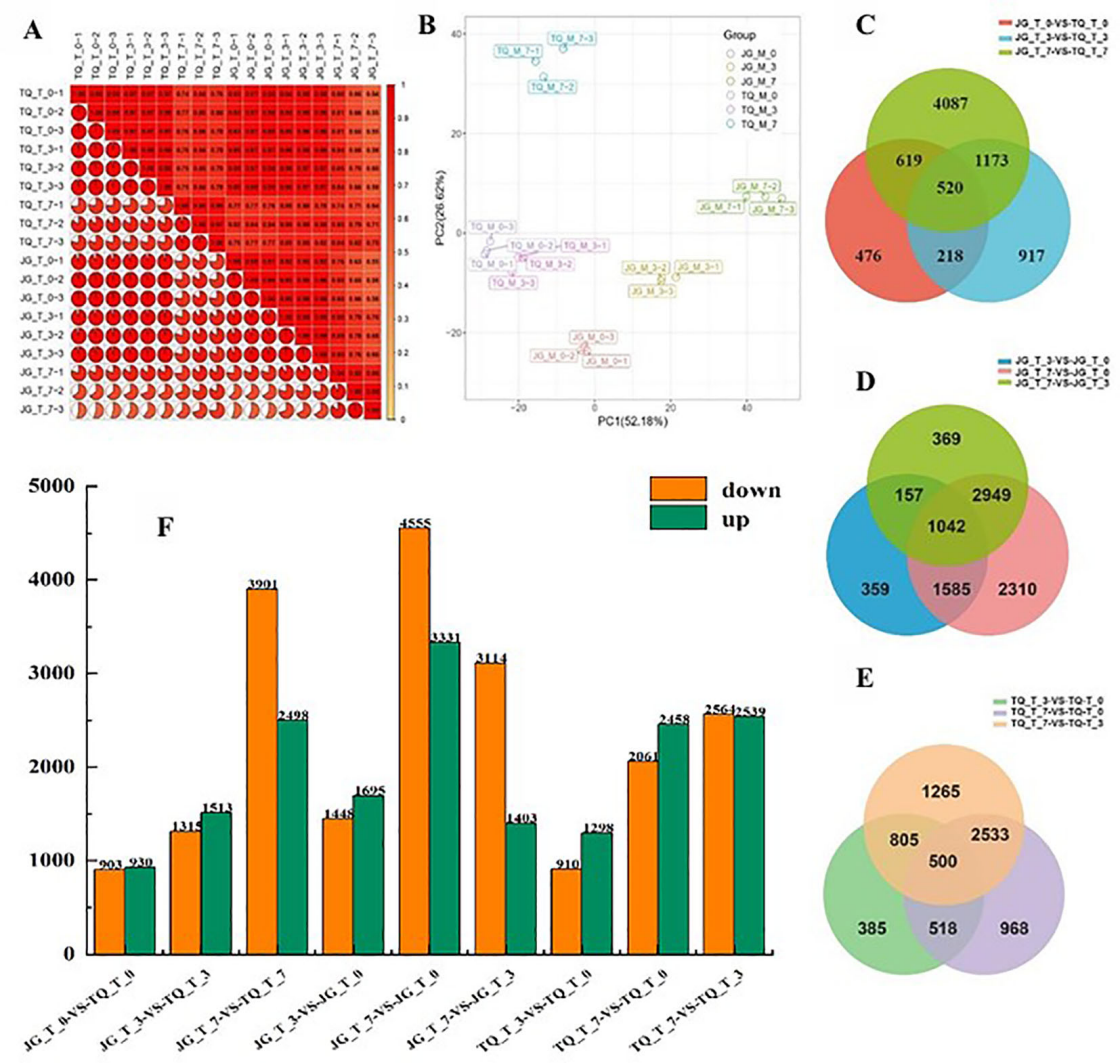
Correlation analysis, principal component analysis (PCA), and identification of differentially expressed genes (DEGs) of sample transcriptomes. **(A)** Correlation analysis of all samples. **(B)** PCA plot of transcriptome results. **(C)** Venn Diagram of DEGs Among Different Germplasm at the Same Developmental Stage. **(D)** Venn Diagram of DEGs Among Comparison Groups of Different Developmental Stages in JG Germplasm. **(E)** Venn Diagram of DEGs Among Comparison Groups of Different Developmental Stages in TQ Germplasm. F Numbers of DEGs between different comparisons.

To identify saline-alkali tolerance genes, leaves from both materials at periods 0, 3, and 7 were subjected to transcriptome sequencing, generating a total of 122.32 Gb of clean data with an average GC content of 45.44%. Using Fold Change ≥ 2 and FDR < 0.05 as criteria, correlation analysis was performed on sequencing data from 18 samples, with results shown in **Figure 2A**. Upregulated and downregulated DEGs in each comparison group are shown in **Figure 2F**. The JG_T_7-VS-JG_T_0 comparison group had the most DEGs (7,886), while JG_T_0-VS-TQ_T_0 had the fewest (1,833). There were 520 common genes among DEGs in JG_T_0-VS-TQ_T_0, JG_T_3-VS-TQ_T_3, and JG_T_7-VS-TQ_T_7 (**Figure 2C**); 1,042 common genes among JG_T_3-VS-JG_T_0, JG_T_7-VS-JG_T_0, and JG_T_7-VS-JG_T_3 (**Figure 2D**); and 500 common genes among TQ_T_3-VS-TQ_T_0, TQ_T_7-VS-TQ_T_0, and TQ_T_7-VS-TQ_T_3 (**Figure 2E**).

#### 3.2.1 GO annotation and KEGG analysis of differentially expressed genes

**Figure 3:**
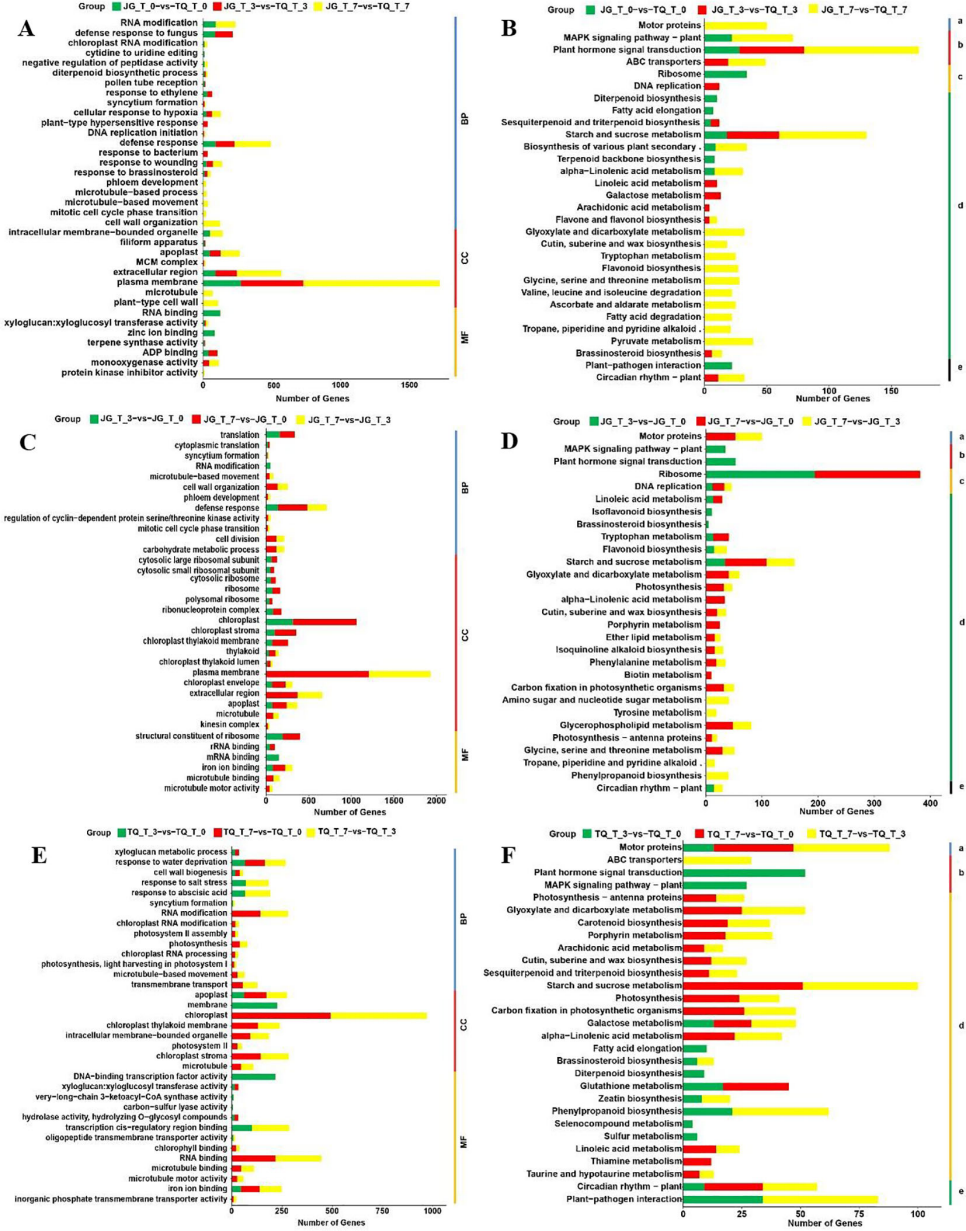
GO **(A)** and KEGG **(B)** annotations of (DEGs) among distinct materials in the same period. GO **(C)** and KEGG **(D)** annotations of (DEGs) in JG material across different periods. GO **(E)** and KEGG **(F)** annotations of (DEGs) in TQ material across different periods. The GO annotations are categorized into three functional groups: BP denotes biological process, CC denotes cellular component, and MF denotes molecular function. The KEGG annotations are divided into five categories: **(A)** represents Cellular Processes, **(B)** represents Environmental Information Processing, **(C)** represents Genetic Information Processing, **(D)** represents Metabolism, and **(E)** represents Organismal Systems.

To analyze the functions of DEGs responsive to saline-alkali stress between JG and TQ materials, GO enrichment analysis and KEGG analysis were performed on DEGs from nine comparison groups. Based on GO annotation (**Figure 3A**), the top 20 GO terms with P < 0.05 in JG_T_0-VS-TQ_T_0, JG_T_3-VS-TQ_T_3, and JG_T_7-VS-TQ_T_7 comparison groups were compared. Annotation terms were mainly concentrated in biological processes, with diterpenoid biosynthetic process, cellular response to hypoxia, defense response, response to wounding, and response to brassinosteroid annotated in all three comparison groups. In cellular components, apoplast, extracellular region, and plasma membrane were annotated in all three comparison groups. In molecular functions, only xyloglucan: xyloglucosyl transferase activity was annotated in all three comparison groups. The highest number of genes were annotated to plasma membrane in the three comparison groups.

Comparing the top 20 KEGG pathways with P < 0.05 annotated in different comparison groups, annotated KEGGs were classified into five categories (**Figure 3B**): Cellular Processes, Environmental Information Processing, Genetic Information Processing, Metabolism, and Organismal Systems, mainly concentrated in Metabolism classification. Plant hormone signal transduction and starch and sucrose metabolism were enriched in all three comparison groups.

GO and KEGG analysis was performed on different time period comparison groups of JG material (JG_T_3-VS-JG_T_0, JG_T_7-VS-JG_T_0, JG_T_7-VS-JG_T_3). Based on GO annotation (**Figure 3C**), syncytium formation and defense response in biological processes were annotated in all three comparison groups; thylakoid, chloroplast envelope, and apoplast in cellular components were annotated in all three comparison groups; iron ion binding in molecular functions was annotated in all three comparison groups.

Comparing the top 20 KEGG pathways with P < 0.05 annotated in different comparison groups, most KEGG pathways annotated in the JG_T_7-VS-JG_T_0 comparison group (**Figure 3D**) were also annotated in the JG_T_7-VS-JG_T_3 comparison group, especially in the Metabolism classification. Starch and sucrose metabolism pathway was annotated in all three comparison groups.

GO and KEGG analysis was performed on different time period comparison groups of TQ material (TQ_T_3-VS-TQ_T_0, TQ_T_7-VS-TQ_T_0, TQ_T_7-VS-TQ_T_3). Based on GO annotation (**Figure 3E**), response to water deprivation and cell wall biogenesis in biological processes were annotated in all three comparison groups; apoplast in cellular components was annotated in all three comparison groups; iron ion binding in molecular functions was annotated in all three comparison groups.

Analyzing the top 20 KEGG annotation results with P < 0.05 for TQ material, annotated KEGGs were classified into four categories (**Figure 3F**): Cellular Processes, Environmental Information Processing, Metabolism, and Organismal Systems. In the Metabolism classification, KEGG pathways annotated in the TQ_T_7-VS-TQ_T_0 comparison group were also annotated in the TQ_T_7-VS-TQ_T_3 comparison group. In the Cellular Processes classification, motor proteins were annotated in all three comparison groups. In the Metabolism classification, galactose metabolism was annotated in all three comparison groups. In the Organismal Systems classification, circadian rhythm-plant was annotated in all three comparison groups.

#### 3.2.2 Expression characteristics of transcription factors in saline-alkali stress response

**Figure 4:**
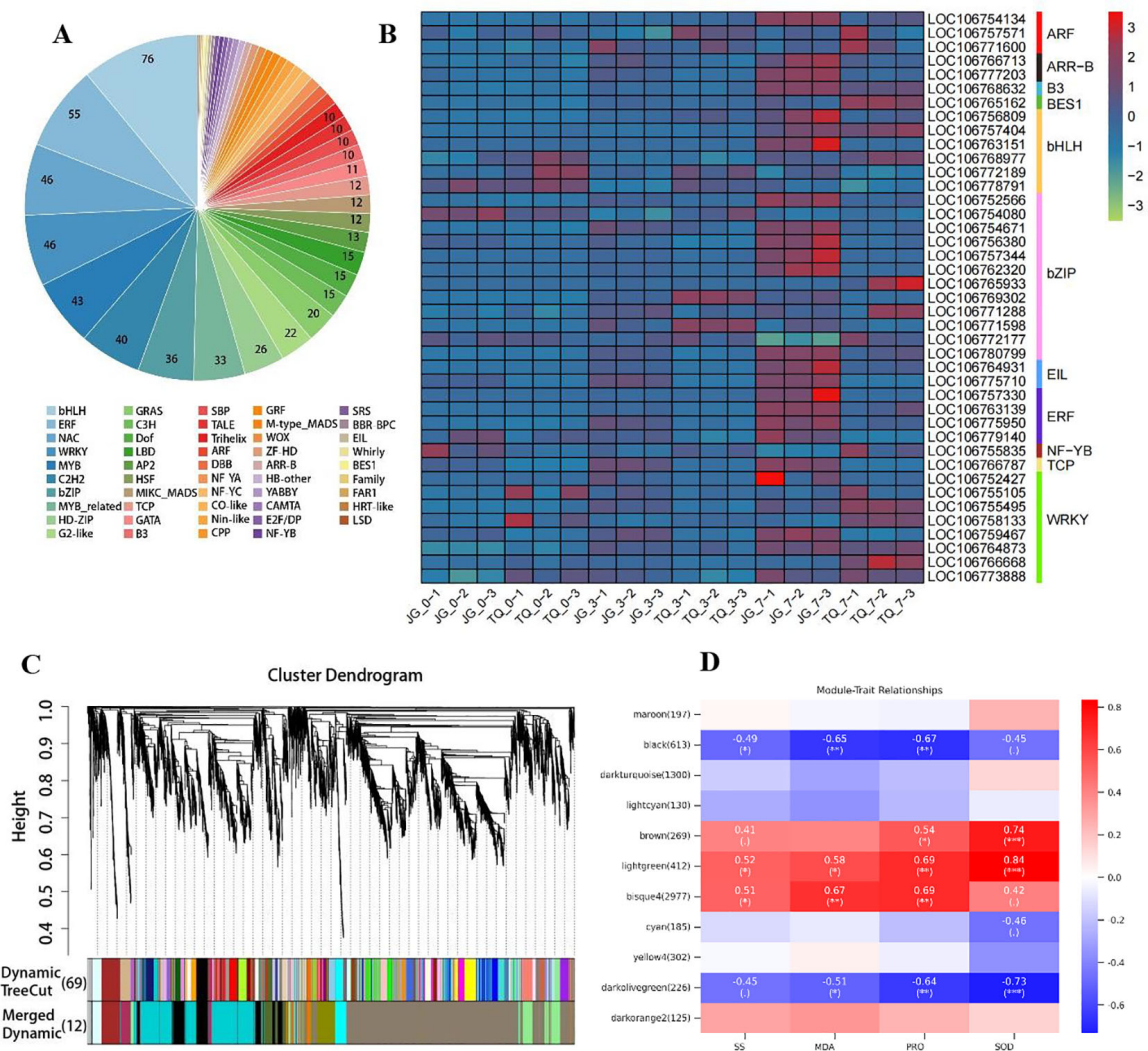
**(A)** Classification of transcription factor families; **(B)** Clustered heatmap of transcription factor expression in all samples. **(C, D)** WGCNA co-expression network and module trait correlation analysis of the samples. In panel **(D)**, asterisks denote statistical significance: * represents *P* < 0.05, ** represents *P* < 0.01.

Transcription factors are important regulators of plant saline-alkali tolerance. In JG and TQ same-period comparison groups, transcription factors from the top 15 KEGG pathways with the smallest p-values were visualized (**Figure 4B**). As saline-alkali stress continued, in JG material, expression of transcription factors from EIL, ERF, B3, and ARR-B families increased, WRKY family transcription factor expression showed minimal increase, while expression of *LOC106755853* in the NF-YB family and *LOC106772177* in the bZIP family decreased. In TQ material, with prolonged stress time, expression of WRKY, NF-YB, and BES1 family transcription factors increased significantly, while expression of *LOC106772189* and *LOC106778791* in bHLH decreased. At period 3, transcription factor expression differences were not sufficiently significant. At period 7, it can be seen that JG and TQ accessions often exhibited opposite expression patterns for the same transcription factor.

Transcription factors in the WRKY family synergistically activate key enzymes involved in carbon fixation to sustain photosynthetic efficiency, promote proline synthesis to maintain osmotic homeostasis, and induce antioxidant enzymes to scavenge reactive oxygen species (ROS) and prevent membrane damage. In TQ, insufficient activation of these transcription factors leads to interrupted carbon flux, osmotic imbalance, and oxidative damage. The transcription factor *LOC106764931* in the EIL family acts as a core gene in ethylene signaling, drives proline accumulation and energy metabolic homeostasis, and serves as an upstream switch for the global stress response in JG. In TQ, the weak response of this gene results in disrupted hormonal signal transduction. The bZIP family contains core transcription factors of the ABA signaling pathway, which act in coordination with WRKY to activate the antioxidant system for ROS scavenging and maintain redox homeostasis. They also assist in activating carbon fixation and proline metabolism, thereby strengthening the maintenance of multiple homeostatic processes in JG. In TQ, the low expression level of this family leads to severe oxidative damage (**Table 2**).

**Table 2:** Transcription factor families and their regulated KEGG pathways and metabolites.

TF family	TF	Pathway module	Metabolites
WRKY	LOC106752427 LOC106755495	Carbon fixation in photosynthetic organisms	S7P, Pyr, Pro, MDA
		Arginine and proline metabolism	
		Redox homeostasis	
ERF	LOC106757330 LOC106763139 LOC106775950	Pyruvate metabolism	Pyr, Asp, Mal
		Plant hormone signal transduction	
		Glyoxylate and dicarboxylate metabolism	
EIL	LOC106764931	Arginine and proline metabolism	Pro, Asp, Mal
		Pyruvate metabolism	
		Plant hormone signal transduction	
bZIP	LOC106757344 LOC106754671 LOC106762320 LOC106765933	Arginine and proline metabolism, Redox homeostasis	Pro, SOD, POD, MDA

#### 3.2.3 WGCNA analysis of differentially expressed genes

To screen key genes playing crucial roles in mungbean saline-alkali tolerance, co-expression network analysis was performed on DEGs from samples at three time periods (**Figure 4D**). All genes were divided into 12 modules, with module gene numbers ranging from 121 to 2,977. Four modules showed significant correlation with traits: The bisque4 and light green modules were positively correlated with the traits, characterized by high SOD activity, high Pro content, and low MDA content, thereby maintaining cellular homeostasis. In contrast, the dark olive green and black modules were negatively correlated with the traits, showing low SOD activity, low Pro content, and high MDA content, which disrupt cellular homeostasis.

#### 3.2.4 qRT-PCR validation of key genes

**Figure 5:**
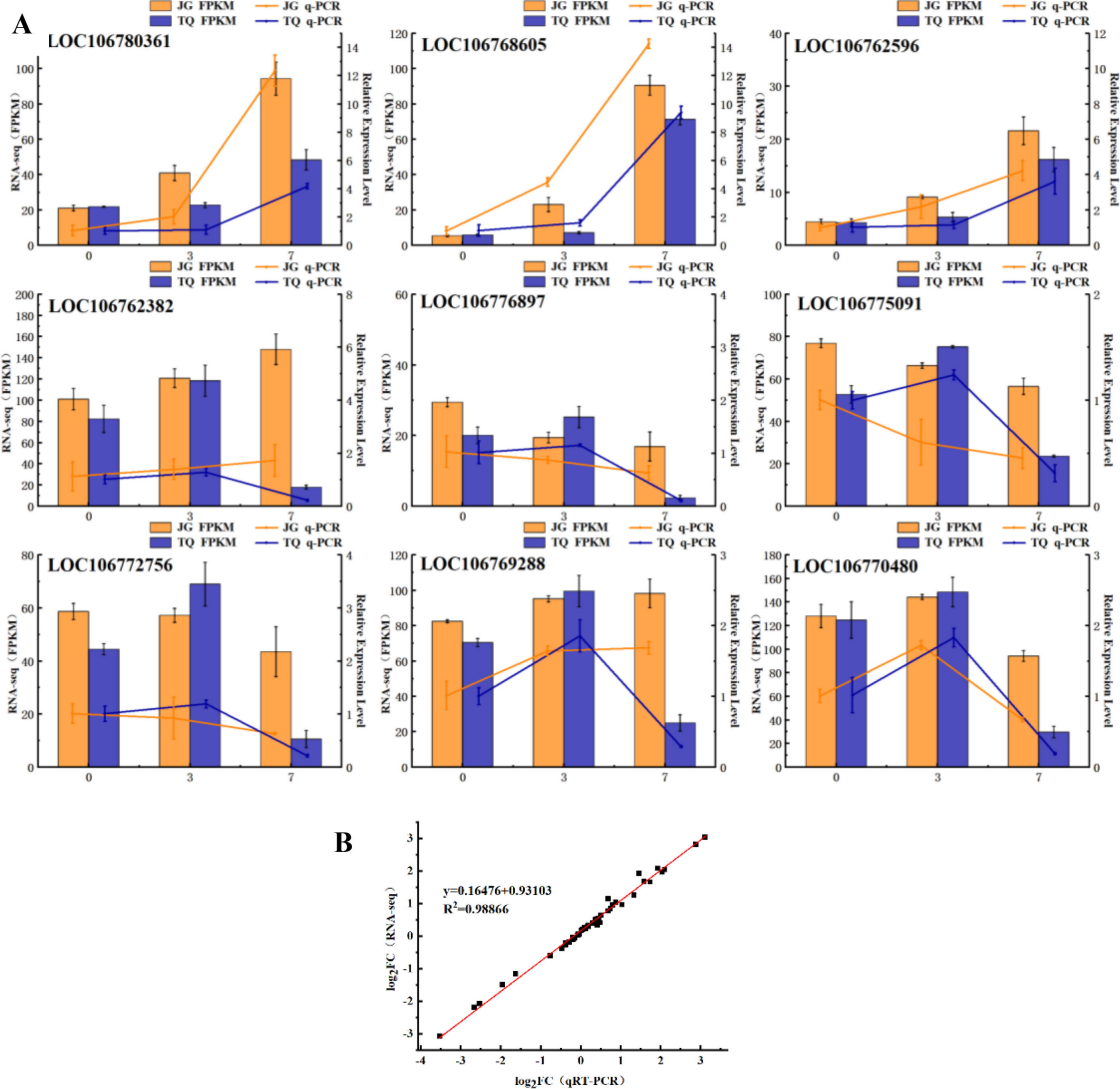
**(A)** qRT-PCR validation of DEGs, **(B)** Linear fitting plot.

Based on WGCNA and KEGG enrichment analysis, 9 genes with significant expression differences were selected for qRT-PCR validation. The results showed that transcriptome sequencing results were largely consistent with qRT-PCR results, verifying the reliability of transcriptome analysis data (**Figure 5A**). To verify the reliability of the transcriptome data, we performed linear fitting between the qRT-PCR and RNA-Seq data for the above 9 genes (**Figure 5B**).

### 3.3 Metabolome changes in mungbean materials under saline-alkali stress

#### 3.3.1 PCA between samples and differential metabolite analysis

**Figure 6:**
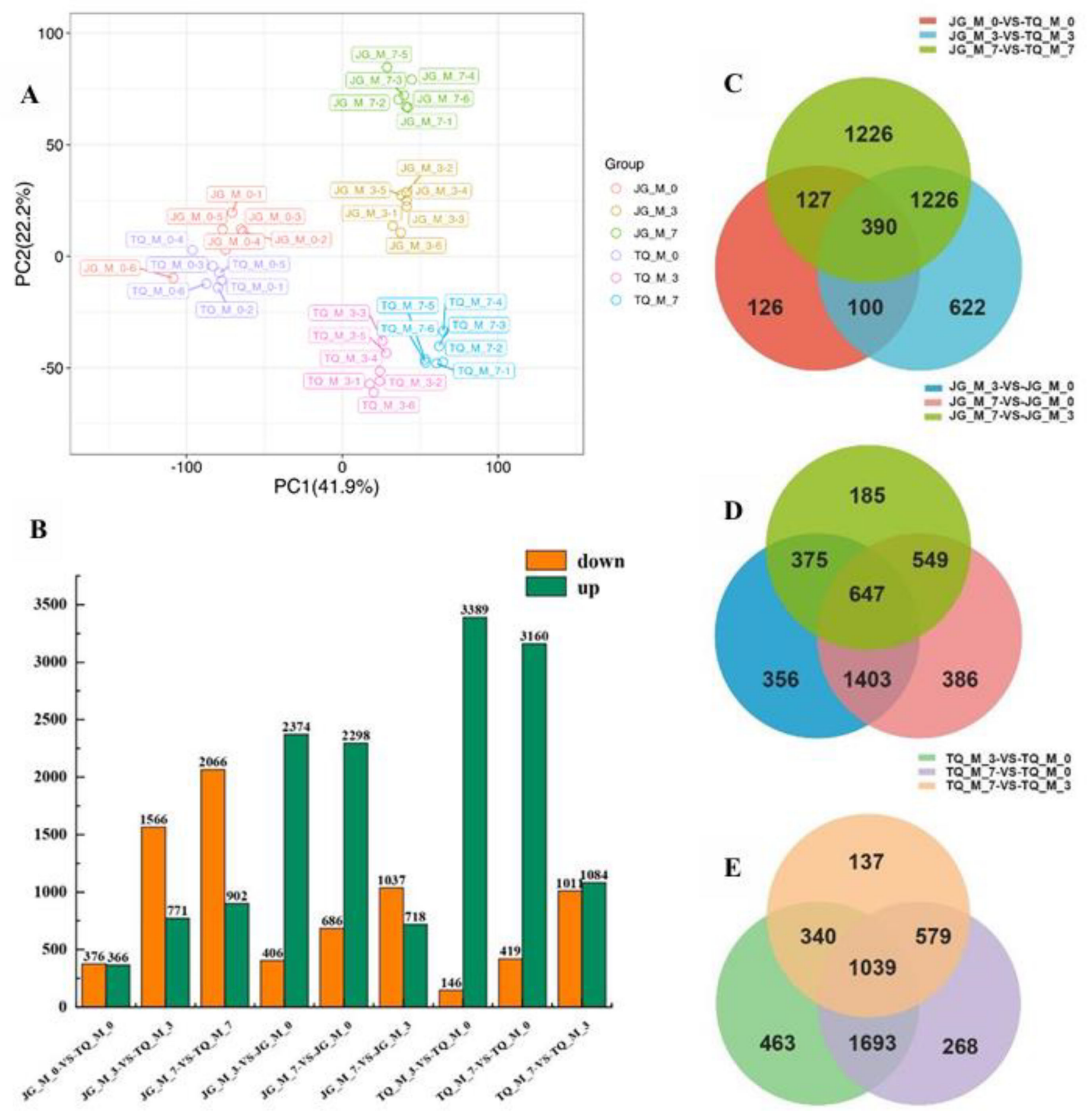
**(A)** PCA plot of metabolomics results. **(B)** Numbers of DAMs between different comparisons. **(C)** Venn Diagram of DAMs Among Different Germplasm at the Same Developmental Stage. **(D)** Venn Diagram of DAMs Among Comparison Groups of Different Developmental Stages in JG Germplasm. **(E)** Venn Diagram of DAMs Among Comparison Groups of Different Developmental Stages in TQ Germplasm.

To comprehensively analyze mungbean response to saline-alkali stress, leaves from both materials at periods 0, 3, and 7 were subjected to non-targeted metabolome sequencing, detecting a total of 5,905 metabolites. The results showed that 100% of the metabolites had RSD < 30%, satisfying the quality control criteria for untargeted metabolomics. To determine different metabolic products in JG and TQ materials under saline-alkali stress, principal component analysis was used to analyze mungbean material metabolic profiles. Results showed clear separation of mungbean metabolic profiles at three time periods (**Figure 6A**), with metabolome data principal component analysis showing stable consistency with transcriptome data principal component analysis. The OPLS-DA model achieved R2Y = 0.992 and Q2 = 0.926. A 200-permutation test confirmed no overfitting of the model, and clear separation was observed between groups (**Supplementary Figure S2**). Using FC ≥ 2 or FC < 0.5 and P-value < 0.05 as differential metabolite screening criteria, comparison of different materials at the same period showed more upregulated differential metabolites, while comparison of different periods of the same material showed more downregulated differential metabolites, as shown in **Figure 6B**.

#### 3.3.2 Analysis of differential metabolites in each comparison group

A total of 390 specific differential metabolites were detected among comparison groups of different materials at the same period (**Figure 6C**), 647 specific differential metabolites were detected among comparison groups of different periods of JG material (**Figure 6D**), and 1,039 specific differential metabolites were detected among comparison groups of different periods of TQ material (**Figure 6E**).

#### 3.3.3 Time series trend analysis of differential metabolites

To clearly display changes in differential metabolites across time gradients, time series trend analysis was performed on differential metabolites, identifying 15 clusters (**Supplementary Figure S3**). Metabolites in Clusters 2, 4, and 5 showed increased abundance at period 3 and lesser increase at period 7 in both JG and TQ under saline-alkali stress. Metabolites in Clusters 12, 13, and 15 showed continuous increase in both JG and TQ as saline-alkali stress continued. Metabolites in Clusters 3 and 14 showed opposite trends in JG and TQ under saline-alkali stress.

#### 3.3.4 Analysis of key metabolites

**Figure 7:**
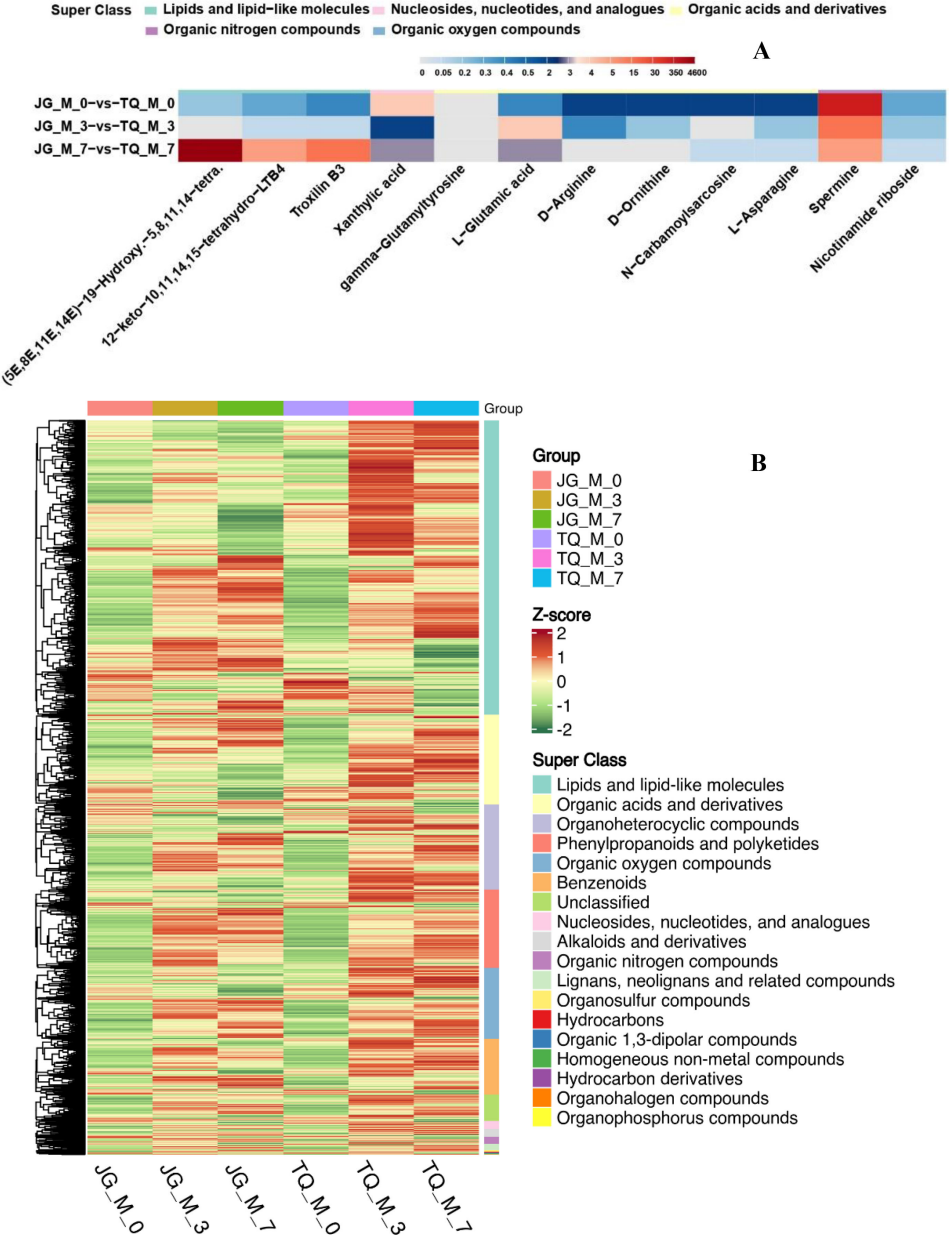
**(A)** Heatmap of key metabolites in JG and TQ materials in response to saline-alkali treatment. **(B)** Clustering heatmap of metabolites in all samples.

Analysis of key metabolites in JG_M_0-VS-TQ_M_0, JG_M_3-VS-TQ_M_3, and JG_M_7-VS-TQ_M_7 (**Figure 7A**) identified 12 products with significant metabolic differences, classified into five categories. D-arginine, D-ornithine, L-asparagine, spermine, and nicotinamide riboside were downregulated with continued stress. (5E,8E,11E,14E)-19-hydroxyicosa-5,8,11,14-tetraenoic acid, 12-keto-10,11,14,15-tetrahydro-LTB4, and troxilin B3 showed significant upregulation. Gamma-glutamyl tyrosine showed no significant changes.

#### 3.3.5 Correlation clustering analysis of metabolites

Through correlation clustering analysis of metabolites detected in JG and TQ materials, metabolites were grouped and their expression under different saline-alkali stress treatments was visualized in a heatmap (**Figure 7B**). Results showed that detected substances exhibited different upregulation or downregulation trends in JG and TQ materials at different treatment periods. Most differential metabolites belonged to lipids and lipid-like molecules and organic acids and derivatives, with TQ material showing more pronounced metabolite upregulation after saline-alkali stress.

#### 3.3.6 KEGG analysis of differential metabolites

**Figure 8:**
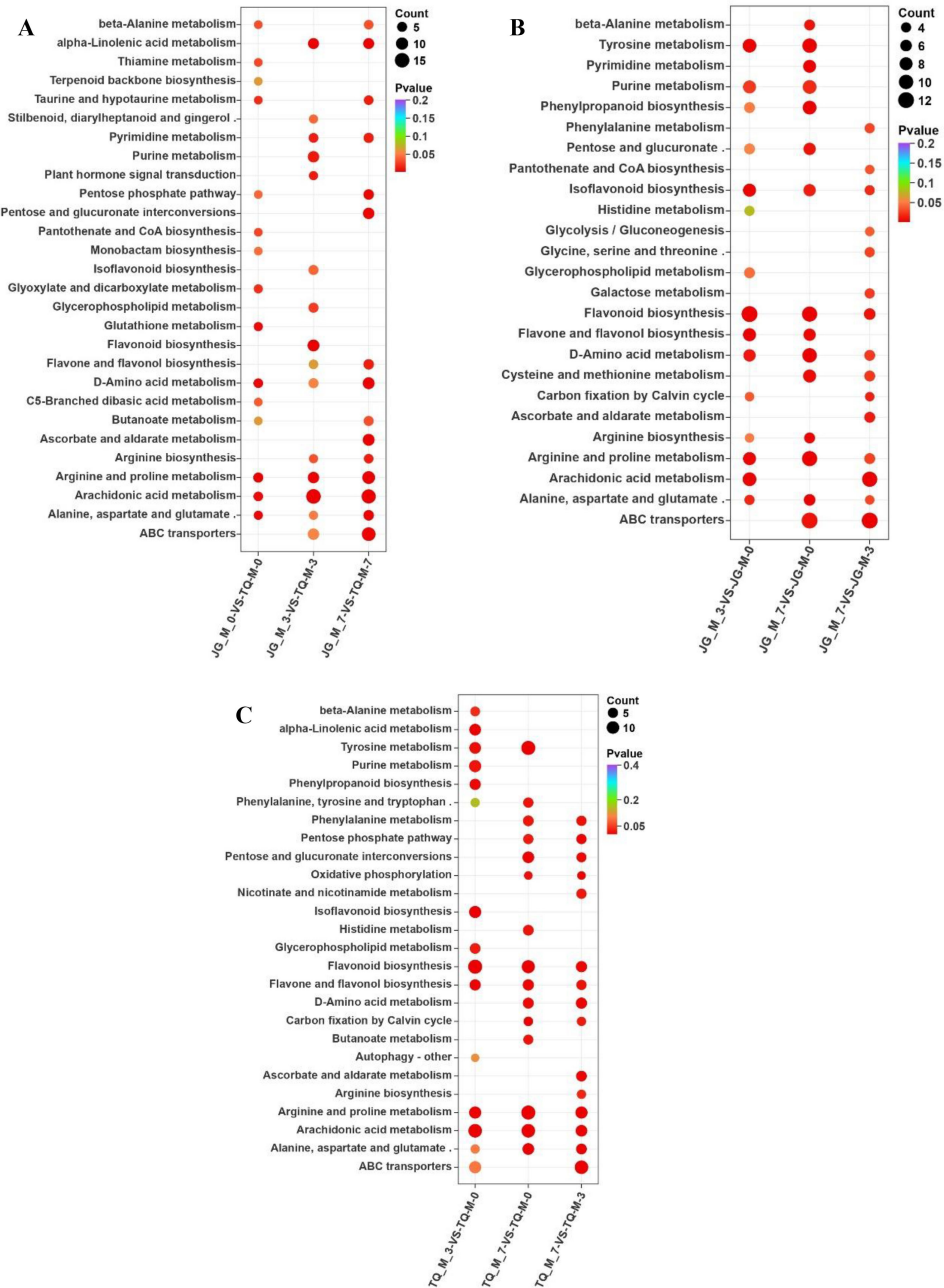
**(A)** KEGG analysis of DAMs in different materials during the same period. **(B)** KEGG analysis of differential metabolites in JG material during different periods. **(C)** KEGG analysis of differential metabolites in TQ material during different periods.

KEGG pathway enrichment analysis was performed on differential metabolites from different materials at the same period (**Figure 8A**). Comparing the top 15 enriched metabolic pathways in JG_M_0-VS-TQ_M_0, JG_M_3-VS-TQ_M_3, and JG_M_7-VS-TQ_M_7, pathways enriched in all three comparison groups included D-amino acid metabolism, arginine and proline metabolism, and arachidonic acid metabolism, with the highest number of differential metabolites annotated in the arachidonic acid metabolism pathway in JG_M_3-VS-TQ_M_3 and JG_M_7-VS-TQ_M_7.

Comparing the top 15 enriched metabolic pathways in JG_M_3-VS-JG_M_0, JG_M_7-VS-JG_M_0, and JG_M_7-VS-JG_M_3 (**Figure 8B**), pathways enriched in all three comparison groups included isoflavonoid biosynthesis, flavonoid biosynthesis, D-amino acid metabolism, arginine and proline metabolism, and alanine, aspartate and glutamate metabolism, with the highest number of differential metabolites annotated in the ABC transporters pathway in JG_M_7-VS-JG_M_3.

Comparing the top 15 enriched metabolic pathways in TQ_M_3-VS-TQ_M_0, TQ_M_7-VS-TQ_M_0, and TQ_M_7-VS-TQ_M_3 (**Figure 8C**), pathways enriched in all three comparison groups included flavonoid biosynthesis, flavone and flavonol biosynthesis, arginine and proline metabolism, arachidonic acid metabolism, and alanine, aspartate and glutamate metabolism, with the highest number of differential metabolites annotated in the arginine and proline metabolism pathway in TQ_M_7-VS-TQ_M_0.

Among all annotated metabolic pathways, arginine and proline metabolism was significantly enriched in every comparison group, while flavonoid biosynthesis and alanine, aspartate and glutamate metabolism pathways were significantly enriched in all comparison groups of the same material at different periods.

### 3.4 Integrated analysis of transcriptome and metabolome

#### 3.4.1 Correlation analysis of DEGs and differential metabolites

For the two materials, the top 30 significantly differential items were selected from the transcriptome and metabolome, respectively. Differentially expressed genes and differential metabolites were screened therefrom, and the correlation between them was calculated, as shown in the **Supplementary Figures S4**, **S5**.

#### 3.4.2 Integrated pathway enrichment analysis

**Figure 9:**
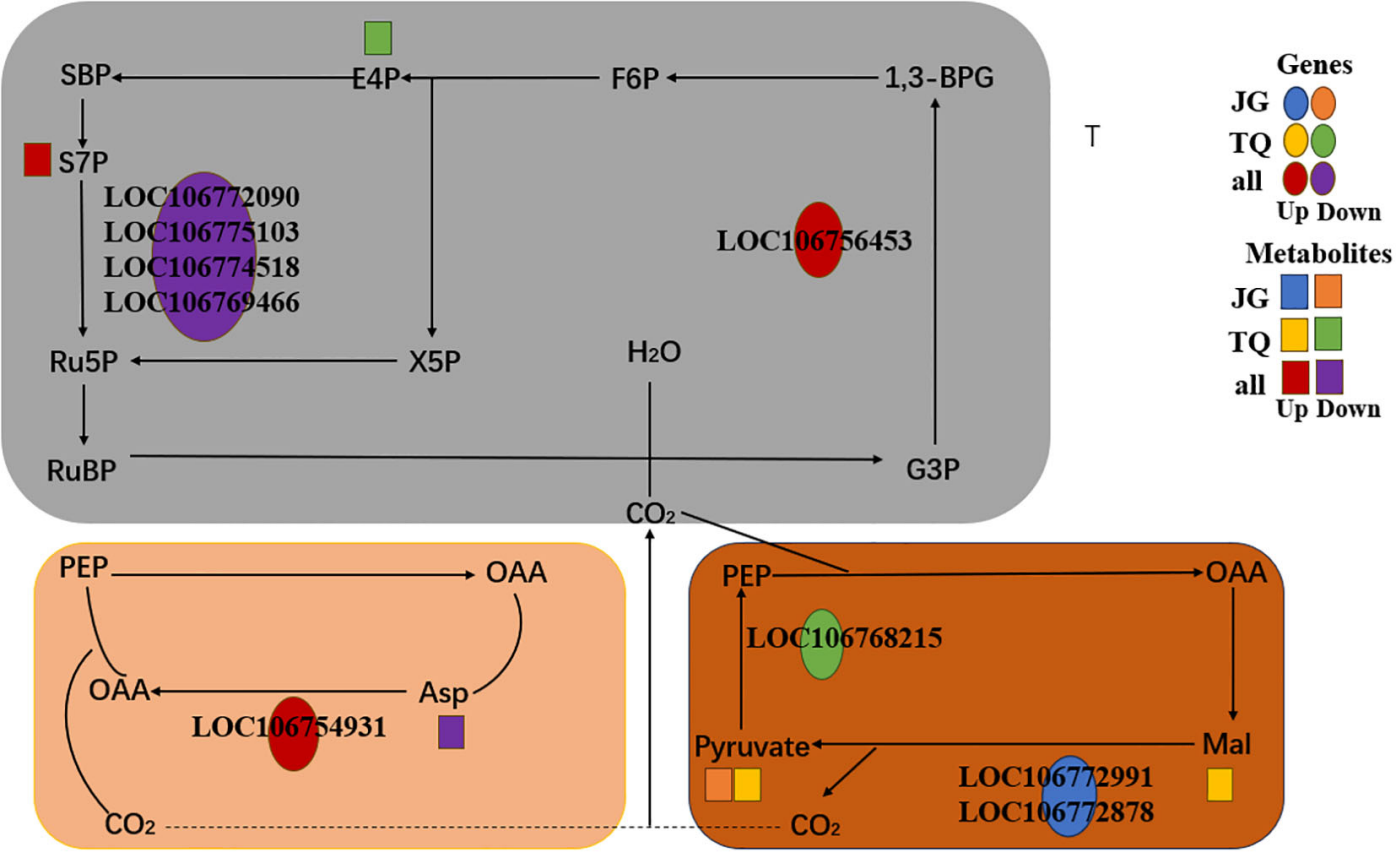
Pathway map of carbon fixation in photosynthetic organisms in JG and TQ materials under saline-alkali stress.

Co-enrichment analysis was performed on the DEGs and differentially accumulated metabolites of JG and TQ materials at stage 7 and stage 0, among which carbon fixation in photosynthetic organisms was significantly co-enriched in both materials, indicating that this metabolic pathway plays a crucial role in the response to saline-alkali stress (**Supplementary Figure S6**). To understand genes encoding key enzymes and participating in important compound synthesis or degradation under saline-alkali stress, DEGs and differential metabolites produced after saline-alkali stress in JG and TQ materials were mapped to the carbon fixation in photosynthetic organisms pathway (**Figure 9**), providing more information on this metabolic pathway’s response to saline-alkali stress. Sedoheptulose-7P content significantly increased in both materials after stress. As a core intermediate metabolite of the Calvin cycle and pentose phosphate pathway, its content changes may be directly related to dynamic regulation of carbon skeleton rearrangement and secondary metabolite precursor supply, serving as an important marker metabolite of photosynthetic carbon metabolism system response under saline-alkali stress (Wang et al., 2024). Aspartate content decreased in both materials, possibly involving reprogramming of amino acid metabolic networks or adaptive adjustment of osmotic regulation systems, further suggesting it as a common response marker for saline-alkali stress (Jia et al., 2019). Pyruvate content decreased in JG material but increased in TQ material, with this difference possibly stemming from divergence in regulatory patterns in glycolysis/gluconeogenesis pathways between the two. Erythrose-4P content decreased in TQ material with no significant change in JG material, reflecting TQ material’s greater sensitivity to saline-alkali stress in carbon flow distribution in the pentose phosphate pathway. Malate increased in TQ material with no significant difference in JG material, indicating that saline-alkali stress reduces photosynthetic capacity and primary metabolite decomposition efficiency in TQ material.

**Supplementary Figure S7** shows expression of common genes enriched in the carbon fixation in photosynthetic organisms pathway in JG and TQ materials. According to KEGG annotation, *LOC106754509* and *LOC106772717* genes encode fructose-1,6-bisphosphatase, participating in important sugar metabolism pathways such as glycolysis/gluconeogenesis and pentose phosphate, helping plants synthesize glucose from non-sugar substances, enhancing plant stress resistance while participating in carbon metabolism processes, regulating carbon fixation in plant photosynthesis, and promoting synthesis and distribution of photosynthetic products, which is significant for plant growth, development, and substance accumulation. *LOC106769466, LOC106774518, LOC106775103* genes encode ribose-5-phosphate isomerase, providing ribose for nucleic acid synthesis while participating in carbon skeleton rearrangement in sugar metabolism. Aspartate aminotransferase *LOC106754931*, phosphoenolpyruvate carboxylase *LOC106760805*, and phosphoenolpyruvate carboxykinase *LOC106777641* all increased significantly, maintaining plant energy supply through the gluconeogenesis pathway, regulating osmotic balance, enhancing antioxidant capacity, and providing support for mungbean resistance to saline-alkali stress (Dunning et al., 2019).

To explore differences in metabolite changes in the carbon fixation metabolic pathway in photosynthetic organisms between JG and TQ materials, expression pattern analysis was performed on their specific genes (**Supplementary Figure S8**). Sedoheptulose-7P content significantly increased in both JG and TQ, possibly because transketolase *LOC106772090* and ribulose-5-phosphate isomerase *LOC106775103, LOC106774518, LOC106769466*, which catalyze conversion to the subsequent product ribose-5-phosphate, showed significantly decreased transcription levels after stress, inhibiting conversion of sedoheptulose-7P to ribose-5-phosphate and reducing NADPH, weakening mungbean antioxidant damage resistance. Aspartate content significantly decreased in both JG and TQ, possibly because aspartate aminotransferase *LOC106754931* showed significantly increased transcription levels after stress. To resist saline-alkali stress, mungbean accelerated conversion of aspartate to oxaloacetate, which can increase energy supply under stress and maintain normal plant growth.

Malate is catalyzed by malic enzyme to produce pyruvate. In TQ material during this process, malic enzymes *LOC106762185, LOC106759749* showed significantly decreased expression after stress, leading to inhibited malate catalysis and significantly increased malate content. In JG material during this process, malic enzymes *LOC106772991, LOC106772878* showed significantly increased expression after stress, ensuring malic enzyme catalytic capacity, allowing the reaction to proceed normally, preventing malate accumulation with no significant content change. NADPH generated during malate to pyruvate conversion can support the antioxidant system in clearing reactive oxygen species, providing reducing power for secondary metabolite synthesis, and enhancing mungbean saline-alkali tolerance.

Pyruvate content increased in TQ material, possibly because the key gene *LOC106768215* in phosphoenolpyruvate carboxykinase showed decreased expression after saline-alkali stress, leading to low catalytic efficiency in subsequent pyruvate reactions, making reactions difficult to proceed and causing pyruvate accumulation. Under saline-alkali stress, plant cell repair, ion balance regulation, and other processes consume dramatically increased energy, promoting pyruvate conversion to acetyl-CoA to enter the tricarboxylic acid cycle for rapid ATP generation. In JG material, the *LOC106768215* gene showed no significant inhibition, but to resist saline-alkali stress, pyruvate consumption accelerated, reducing content.

## 4 Discussion

Mungbean is an important food crop rich in various amino acids, vitamins, and minerals, with a long cultivation history in China (Zhang X. et al., 2026). Due to reduced arable land, minor crops like mungbean must utilize saline-alkali land for cultivation, but saline-alkali environments are unfavorable for mungbean growth (Da et al., 2026). Numerous studies confirm that saline-alkali stress leads to difficult nutrient uptake by mungbean roots, leaf chlorosis, seriously affecting photosynthesis and overall plant growth (Iqbal et al., 2024). This study subjected saline-alkali-tolerant material JG111-6 (JG) and saline-alkali-sensitive material Tuquan mungbean (TQ) to 100 mmol·L^-^¹ mixed saline-alkali stress to explore key genes in mungbean saline-alkali stress response and reveal molecular mechanisms of mungbean saline-alkali tolerance.

Superoxide dismutase activity significantly increases in plants under stress, promptly clearing excess O_2_^-^ to protect cell membrane phospholipids, proteins, DNA, and other macromolecules from attack (Huo et al., 2022). The JG genotype exhibited relatively high superoxide dismutase (SOD) activity at the 0 stage and was stably activated after saline-alkali stress. In contrast, the SOD activity of the TQ genotype passively increased dramatically with prolonged stress duration, representing hysteretic stress compensation under stress-induced damage. The TQ genotype displayed poor regulatory stability and failed to effectively block the attack of superoxide anion (O_2_^-^) on cell membranes. Malondialdehyde is a core indicator substance of stress damage, with content reflecting plant cell membrane lipid peroxidation damage degree (Wang et al., 2023). Proline is an important stress-protective substance, undertaking osmotic regulation, maintaining cell water balance, and clearing reactive oxygen functions (Song et al., 2025). The JG genotype possesses an efficient ion regulation mechanism that reduces intracellular Na^+^ accumulation at the source to mitigate osmotic stress. Proline accumulation in JG is adequate to sustain cell turgor and water balance, thereby enabling active adaptation to saline-alkali stress. In contrast, excessive proline accumulation in the TQ genotype may serve as an indicator of cellular metabolic disorder, in which proline accumulates massively as a metabolic byproduct rather than functioning in active osmotic adjustment. In this study, superoxide dismutase, malondialdehyde, and proline contents were directly related to plant saline-alkali stress. The increase magnitudes in the saline-alkali-sensitive TQ genotype were significantly higher than those in the saline-alkali-tolerant JG genotype, which is consistent with the research results by Liangjie et al (Lv et al., 2022). Soluble sugar content showed no significant differences before and after treatment in both materials, possibly because legume osmotic regulation substances rely more on proline (Kaur et al., 2017), leading to this result.

Before and after saline-alkali treatment, numerous DEGs were found between the two materials. GO enrichment analysis showed that comparison of different materials at the same period revealed these DEGs were mainly related to biological processes such as defense response and cellular response to hypoxia. Comparison of different periods of JG material showed DEGs were mainly related to cellular processes such as chloroplast envelope and apoplast. Comparison of different periods of TQ material showed DEGs were mainly concentrated in molecular functions such as iron ion binding and RNA binding. Apoplast was annotated in all comparison groups, and iron ion binding was annotated in all comparison groups of different periods of both materials. KEGG enrichment analysis showed that comparison of different materials at the same period revealed DEGs were mainly enriched in plant hormone signal transduction and starch and sucrose metabolism pathways. Plant hormone signal pathways such as abscisic acid and auxin pathways are core regulatory networks for plants responding to abiotic stress, coordinately regulating physiological processes such as ion homeostasis and osmotic regulation (Deslandes and Rivas, 2012). After stress, the key genes for ABA biosynthesis (NCED, ZEP), receptor genes (PYR/PYL), negative regulators (PP2C), and downstream kinase genes (SnRK2) were all significantly upregulated in both genotypes, and the upregulation magnitude in JG was notably higher than that in TQ. This pathway can activate downstream transcription factors such as bZIP and WRKY to counteract saline-alkali stress. Ethylene signal transduction genes (EIL) and the downstream ERF transcription factor family exhibited significant responses to stress, acting as a critical hub connecting hormone signaling and downstream metabolic pathways. This pathway has also been identified in maize under salt treatment, which further reveals the critical role of signaling pathways in plant responses to saline-alkali stress (Wu et al., 2018). Enhanced starch and sucrose metabolism can provide materials with sufficient energy and osmotic protective substances, maintaining physiological stability under long-term stress (Zhang et al., 2025). In rice, both salt-tolerant and salt-sensitive varieties involve starch and sucrose metabolism in their responses to saline-alkali stress, which is consistent with the findings of the present study (Akilan et al., 2025). With prolonged treatment time, more DEGs enriched in these two pathways. In comparison groups of different periods of JG material, the ribosome pathway showed extremely high gene enrichment levels. Ribosomes are core structures of protein translation, and activation of this pathway indicates JG can rapidly enhance synthesis efficiency of stress-related functional proteins (such as ion transport proteins and antioxidant enzymes), thereby constructing an efficient stress adaptation molecular system. Additionally, starch and sucrose metabolism also showed high DEG enrichment, reflecting JG’s coordinated regulation capacity for energy supply and protective substance synthesis, which is also a key molecular basis for its saline-alkali tolerance. In comparison groups of different periods of TQ material, starch and sucrose metabolism was not enriched at period 3 but significantly enriched at period 7. Unlike JG material, this may lead to no carbon allocation at period 3, resulting in weaker early defense systems (Li H. et al., 2023). The number of DEGs enriched in the plant hormone signal transduction pathway of TQ material was also significantly lower than JG material, indicating defects in its hormone regulatory network. This defect makes it difficult to coordinate multi-dimensional stress adaptation processes, thereby exacerbating its saline-alkali sensitivity. During evolution, plants have formed multiple adaptation mechanisms to cope with stress, with many transcription factors playing important roles in adaptation mechanisms (Wang et al., 2008). This study identified several transcription factors responding to saline-alkali stress. WRKY, bZIP, bHLH, ERF, ARF, and other transcription factors may play defensive roles by regulating downstream gene expression. WRKY is one of the largest transcription factor families in plants, participating in abiotic stress responses through plant hormone signal transduction and ROS clearance (Jiang et al., 2017). Overexpression of *AtWRKY25* and *AtWRKY33* in Arabidopsis increased salt tolerance. In apple salt tolerance research, the *MdERF106* gene of the ERF family promoted expression of downstream MdSOS1, thereby promoting Na^+^ efflux (Yu et al., 2020). Researchers also found that bHLH transcription factors regulate plant responses to environmental changes, such as controlling light responses and interacting with circadian clocks (Martínez-García et al., 2000; Matsushika et al., 2002). In this study, *LOC106756809* and *LOC106763151* in the bHLH family showed significantly increased expression in saline-alkali-tolerant materials with minimal expression changes in saline-alkali-sensitive materials, possibly responding to saline-alkali stress by adjusting photosynthesis. In this study, differentially expressed genes (DEGs) were subjected to weighted gene co-expression network analysis (WGCNA) to identify four key co-expression modules underlying saline-alkali stress, thereby transforming large-scale transcriptomic data into six core cellular homeostatic systems, namely redox homeostasis, osmotic homeostasis, ion homeostasis, carbon and energy homeostasis, protein translation, and hormonal signaling. The saline-alkali-tolerant JG genotype maintained comprehensive homeostasis through the coordinated upregulation of genes in positively correlated modules: transcription factors including ERF/WRKY initiated the stress response at the upstream level; genes for superoxide dismutase (SOD) and proline biosynthesis scavenged reactive oxygen species (ROS) and preserved osmotic balance; ion transporter genes prevented Na^+^ toxicity; hub genes governing carbon fixation/pyruvate metabolism secured energy supply; and the ribosome pathway supported efficient synthesis of defense proteins. In contrast, the saline-alkali-sensitive TQ genotype had an excessively high proportion of genes in negatively correlated modules, with insufficient expression and delayed regulation across the six homeostasis axes. This ultimately caused the collapse of cellular homeostasis, resulting in growth inhibition and damage under saline-alkali stress.

Liquid chromatography-tandem mass spectrometry (LC-MS) has high sensitivity and selectivity, enabling broad detection of metabolites in samples. Metabolites are at the terminal end of plant life activities, representing the most direct plant response to external stimuli. Total metabolite numbers in plants reach up to 200,000, making them ideal targets for studying biosynthesis regulation. Numerous differential metabolites were detected between the two genotypes before and after saline-alkali treatment. The contents of various amino acids exhibited a downward trend under long-term stress. As a leguminous crop, mungbean undergoes severely inhibited nodule nitrogen fixation efficiency under saline-alkali stress, leading to an extreme shortage of available nitrogen supply in plants, which ultimately gives rise to the decreasing tendency of amino acid substances. KEGG pathway enrichment analysis of differential metabolites revealed dynamic adaptation processes of metabolic pathways under saline-alkali stress (Sawada and Hirai, 2013). In comparison of different materials at the same period, purine metabolism showed significant enrichment after treatment, indicating plants enhance nucleic acid synthesis and energy metabolism to provide energy support for cellular physiological activities under stress (Watanabe et al., 2010). Glutathione metabolism, flavonoid biosynthesis, and other antioxidant pathways continued throughout all post-treatment periods, indicating plants maintain oxidative stress defense throughout saline-alkali stress to avoid cellular damage from reactive oxygen accumulation (Zhou et al., 2023). Comparison of JG material treatment and control groups showed continuous strengthening of core stress resistance pathways: differential metabolites in tyrosine metabolism, D-amino acid metabolism, and arginine and proline metabolism pathways increased with prolonged treatment time. As precursor substances of hormones and secondary metabolites, amino acids have important research significance in saline-alkali stress (Bi et al., 2025). Fumagalli et al., 2009 studied metabolomics of two rice varieties under high salt stress, finding significant accumulation of amino acid substances, consistent with this study’s results. JG material maintained stable annotation of flavonoid and flavonol biosynthesis pathways at different periods. Combined with their antioxidant functions, this indicates JG material continuously synthesizes antioxidant substances to clear reactive oxygen species and reduce stress damage. This dynamic response pattern is a key guarantee of its saline-alkali tolerance. Comparison of TQ material treatment and control groups also showed annotation to flavonoid biosynthesis and flavone and flavonol biosynthesis metabolic pathways, but with lower numbers of annotated metabolites, and decreasing numbers of annotated substances with time, leading to TQ material’s inability to effectively synthesize antioxidant substances and maintain membrane stability to resist stress, manifesting as saline-alkali sensitivity.

Co-enrichment of transcriptome and metabolome DEGs and differential metabolites showed significant co-enrichment of the carbon fixation in photosynthetic organisms pathway in both materials. Sedoheptulose-7-phosphate (Sedoheptulose-7P) accumulated in both genotypes, which was attributed to the inhibited transcription of key enzymes catalyzing downstream metabolism, resulting in blocked conversion. The genes encoding phosphoenolpyruvate carboxylase (PEPC) and phosphoenolpyruvate carboxykinase (PEPCK) were significantly upregulated in the JG genotype, driving non-photosynthetic CO_2_ fixation. Furthermore, the JG genotype maintained high expression of the genes encoding NADP-malic enzyme under stress, which alleviated oxidative damage to chloroplasts and preserved the structural basis for photosynthetic carbon fixation. *LOC106754509* and *LOC106772717*, which encode fructose-1,6-bisphosphatase, were both downregulated in the two genotypes after stress treatment; this enzyme is the key rate-limiting enzyme for photosynthetic carbon fixation. The decrease in its activity slows the carbon flux rate of the Calvin cycle and reduces futile carbon fixation, thereby lowering the synthesis of photosynthetic products. It actively suppresses plant vegetative growth and redirects the limited carbon and nitrogen sources to stress resistance pathways. The reduction in this enzyme activity represents a common response across mungbean varieties. In contrast; *LOC106754931*, which encodes aspartate aminotransferase, was upregulated in both genotypes under the same treatment. It is therefore hypothesized that fructose-1,6-bisphosphatase and aspartate aminotransferase may serve as specific hub genes in response to saline-alkali stress. Pyruvate, an intermediate of the tricarboxylic acid (TCA) cycle, provides energy supplementation for mungbean under stress, and its content exhibits opposite changing trends in the two genotypes. In the JG genotype, the synthetic pathway is not blocked, and pyruvate is efficiently shunted into the TCA cycle to maintain mitochondrial energy supply, resulting in a reduction in pyruvate content. In contrast, pyruvate accumulates significantly in the TQ genotype. The inhibited expression of the *LOC106768215* gene reduces the catalytic efficiency of subsequent pyruvate metabolism, preventing pyruvate from effectively entering the TCA cycle. Meanwhile, the downregulation of malic enzyme genes (*LOC106762185*, *LOC106759749*) triggers malate accumulation, which further blocks carbon metabolic flux. As a result, cells are forced to divert pyruvate to the fermentation pathway for energy production, which is consistent with the findings of Yang et al (Yang et al., 2022). Through transcriptome and metabolome sequence analysis, this study revealed changes in mungbean genes and metabolites under saline-alkali stress, finding that expression levels of transcription factors, plant hormone signals, and various metabolic products all underwent significant changes, playing important roles in mungbean saline-alkali stress response.

## 5 Conclusion

In this study, a comprehensive omics analysis was conducted on different mung bean materials under saline-alkali stress. Through transcriptomic and metabolomic investigations, the saline-alkali tolerance mechanisms of mung bean materials were revealed, filling the gaps in the molecular basis of saline-alkali tolerance in mung bean. The number of DEGs in JG at the same stage was higher than that in TQ, and the stress-responsive expression of transcription factors was more prominent in JG. The DEGs of both genotypes were significantly enriched in GO functional terms such as defense response, as well as KEGG pathways including ABC transporters and plant hormone signal transduction. A total of 5,905 metabolites were detected in the metabolomic analysis; D-arginine, aspartate and other compounds were identified as key metabolites in response to saline-alkali stress. Genotypic differences existed in the enrichment pathways of differential metabolites: JG showed more stable annotation, while the number of annotated metabolites in TQ was low and displayed a decreasing trend. The DEGs and differential metabolites of both genotypes were co-enriched in the carbon fixation pathway. The contents of key substances and the expression patterns of genes encoding malic enzyme and phosphoenolpyruvate carboxykinase (PEPCK) were genotype-specific. The specific upregulation and stable expression of relevant genes in JG represent an important molecular feature underlying its stronger saline-alkali tolerance.

## Data Availability

The data presented in the study are deposited in the NCBI BioProject repository, accession number PRJNA1468888.
