# Supplementary material for: Integrated transcriptomic and metabolomic identification of the core pathway of photosynthetic carbon fixation in mung bean under saline-alkali stress
Source: Front Plant Sci. 2026 May 29;17:1823453. doi: 10.3389/fpls.2026.1823453 (PMC13259979; doi:10.3389/fpls.2026.1823453)
Supplement: Supplementary file 1 [file SupplementaryFile1.docx]

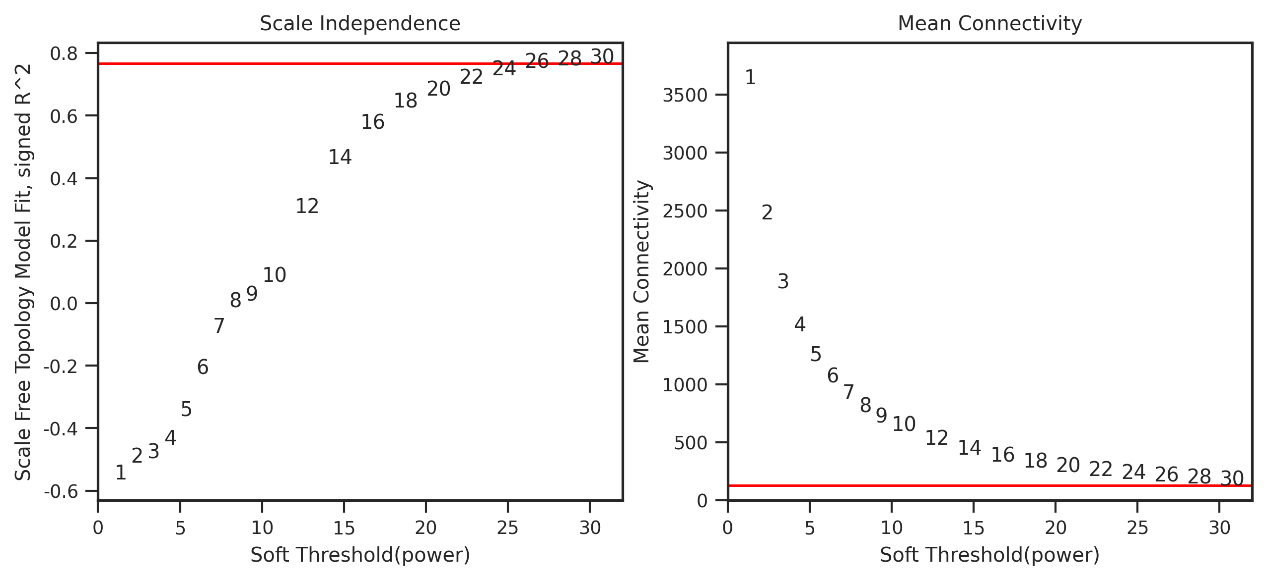


**SFig.1** Scale Independence and Mean Connectivity Plot.


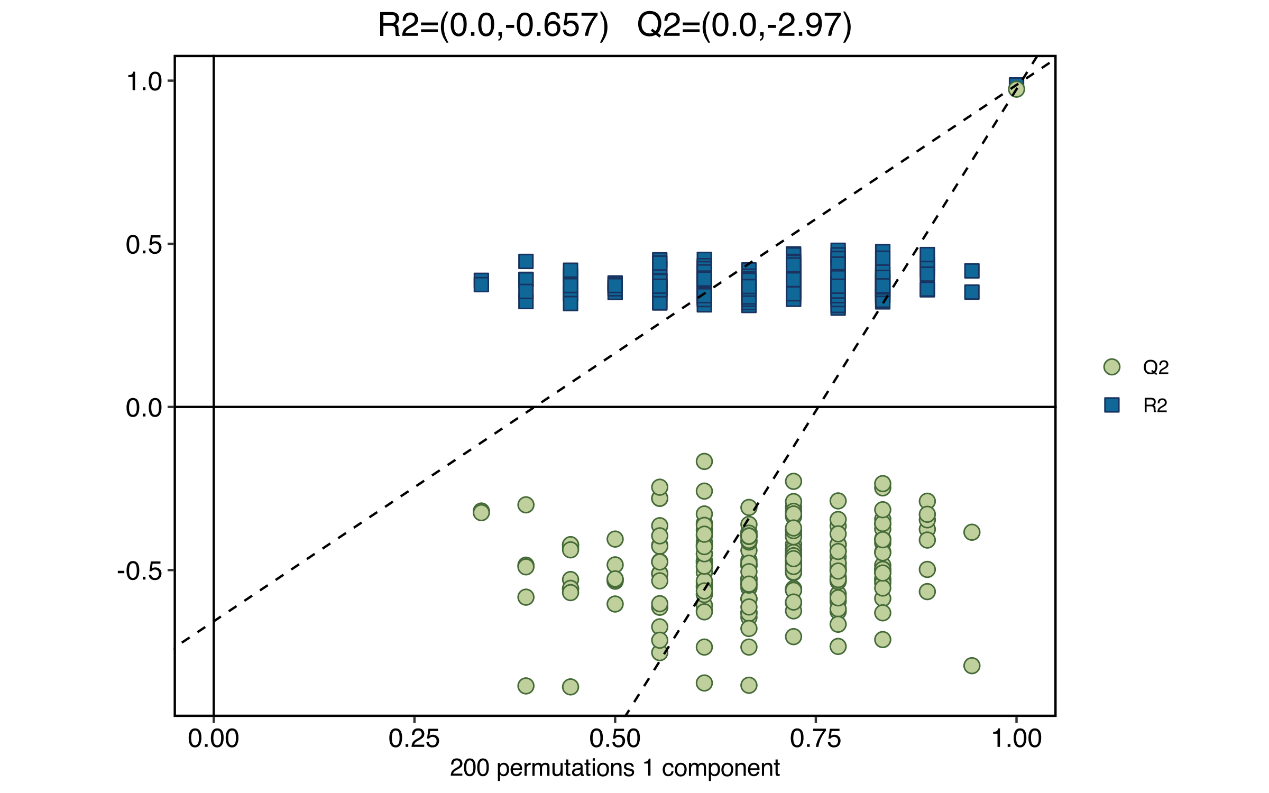


**SFig.2** OPLS-DA Permutation Test Plot


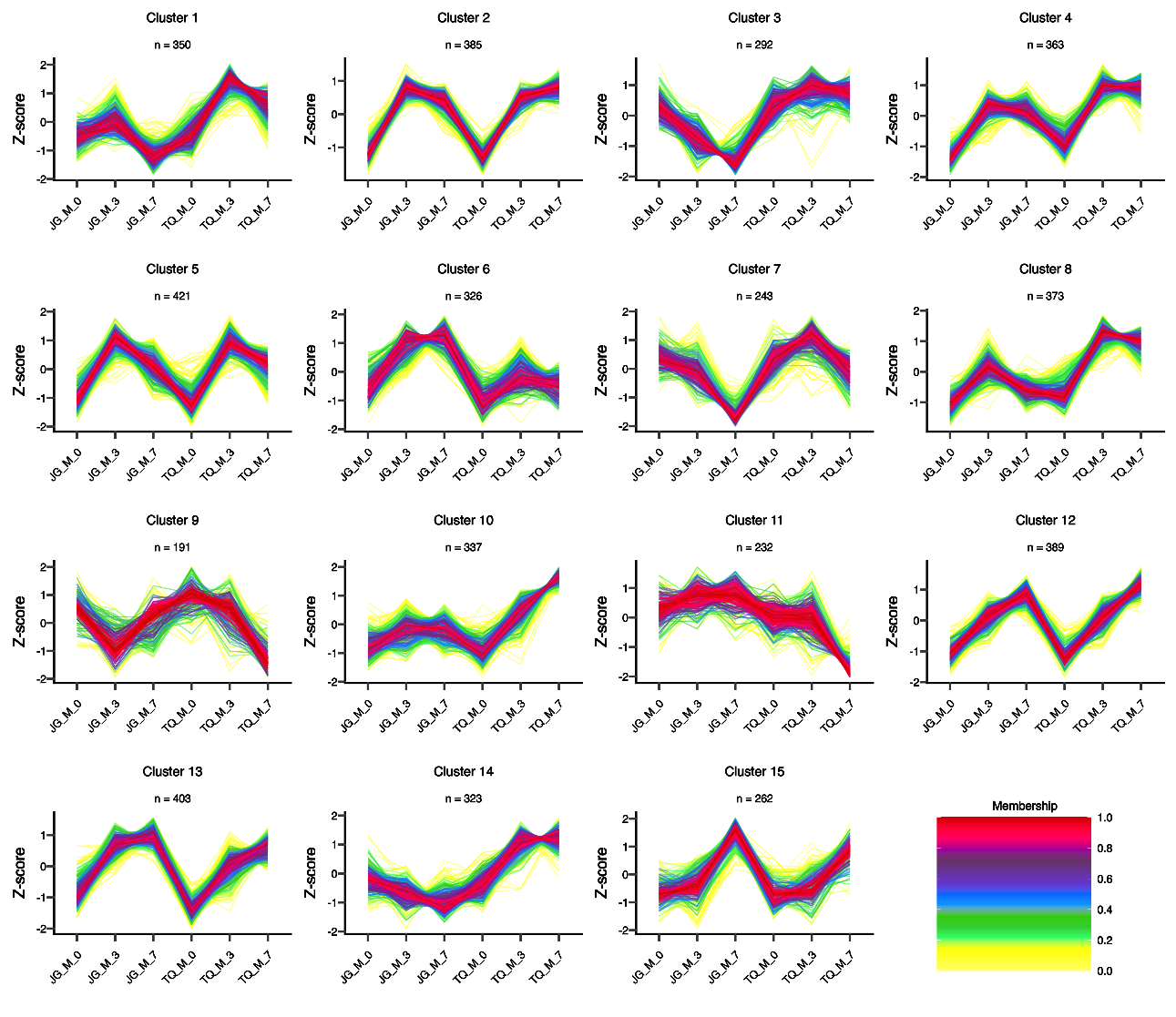


**SFig.3** Time series trend analysis of differential metabolites.


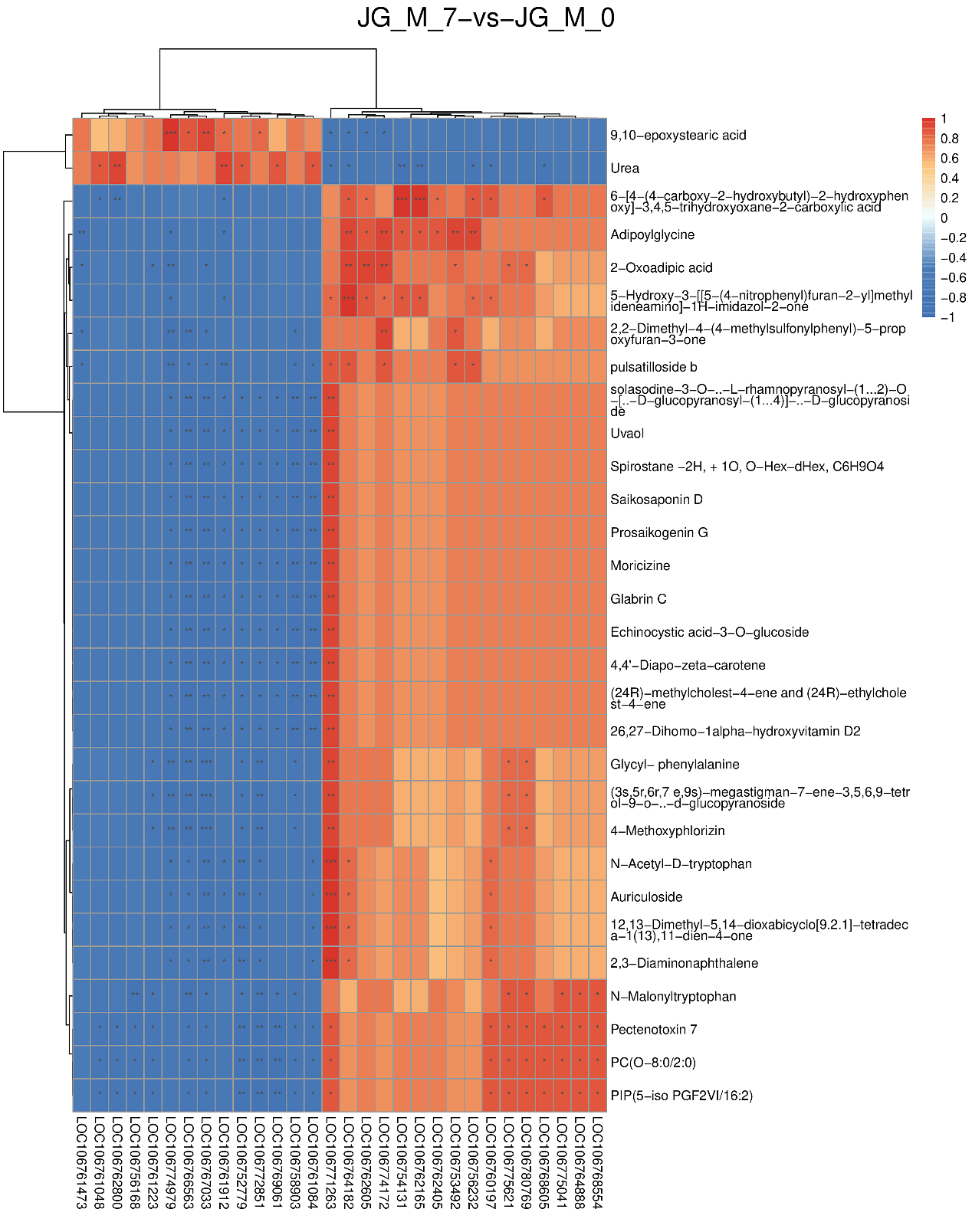


**SFig.4** Correlation analysis between DEGs and DAMs in JG material.

Note: * represents *P* < 0.05, ** represents *P* < 0.01. *** represents *P* < 0.001.


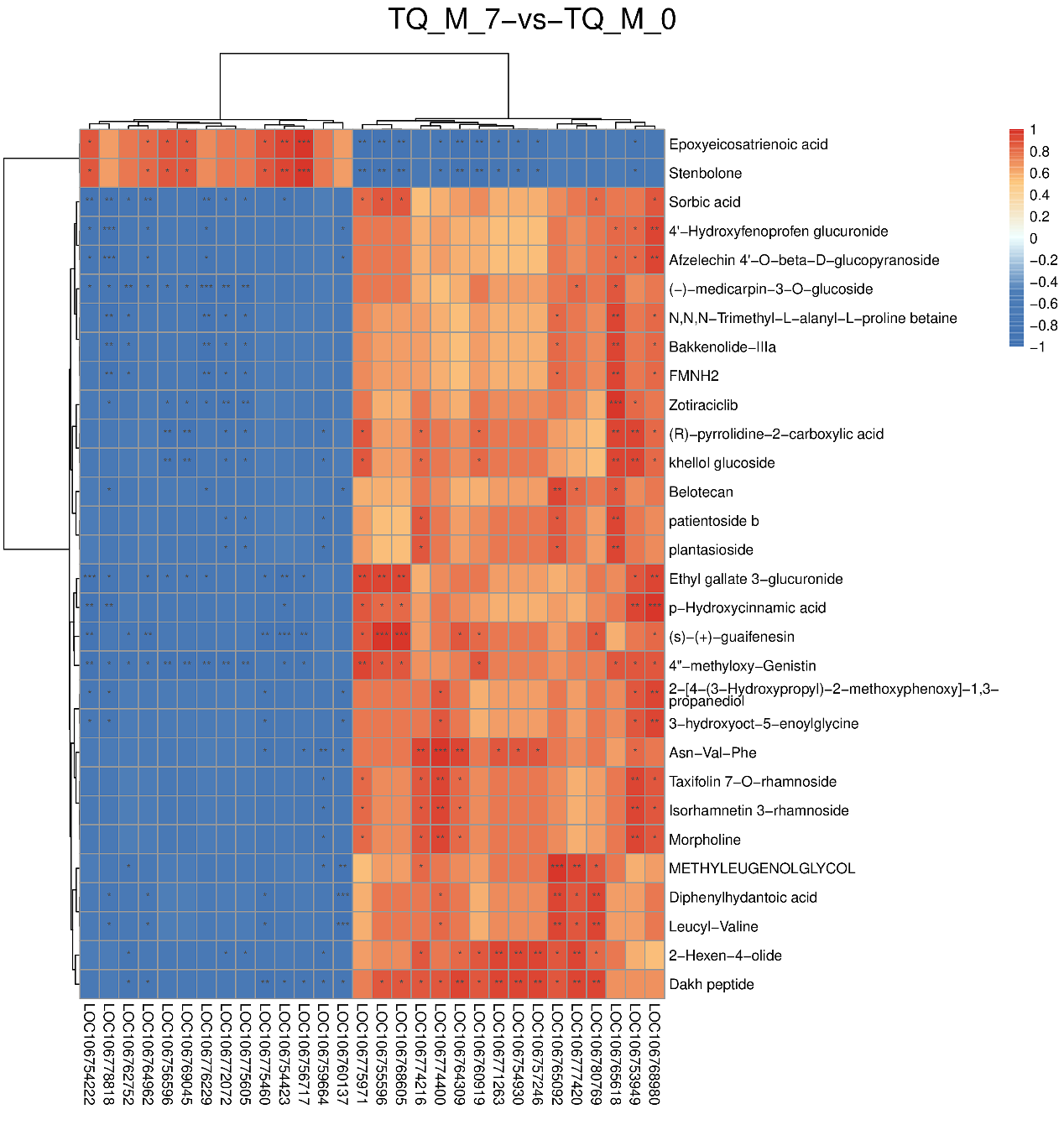


**SFig.5** Correlation analysis between DEGs and DAMs in TQ material.

Note: * represents *P* < 0.05, ** represents *P* < 0.01. *** represents *P* < 0.001.


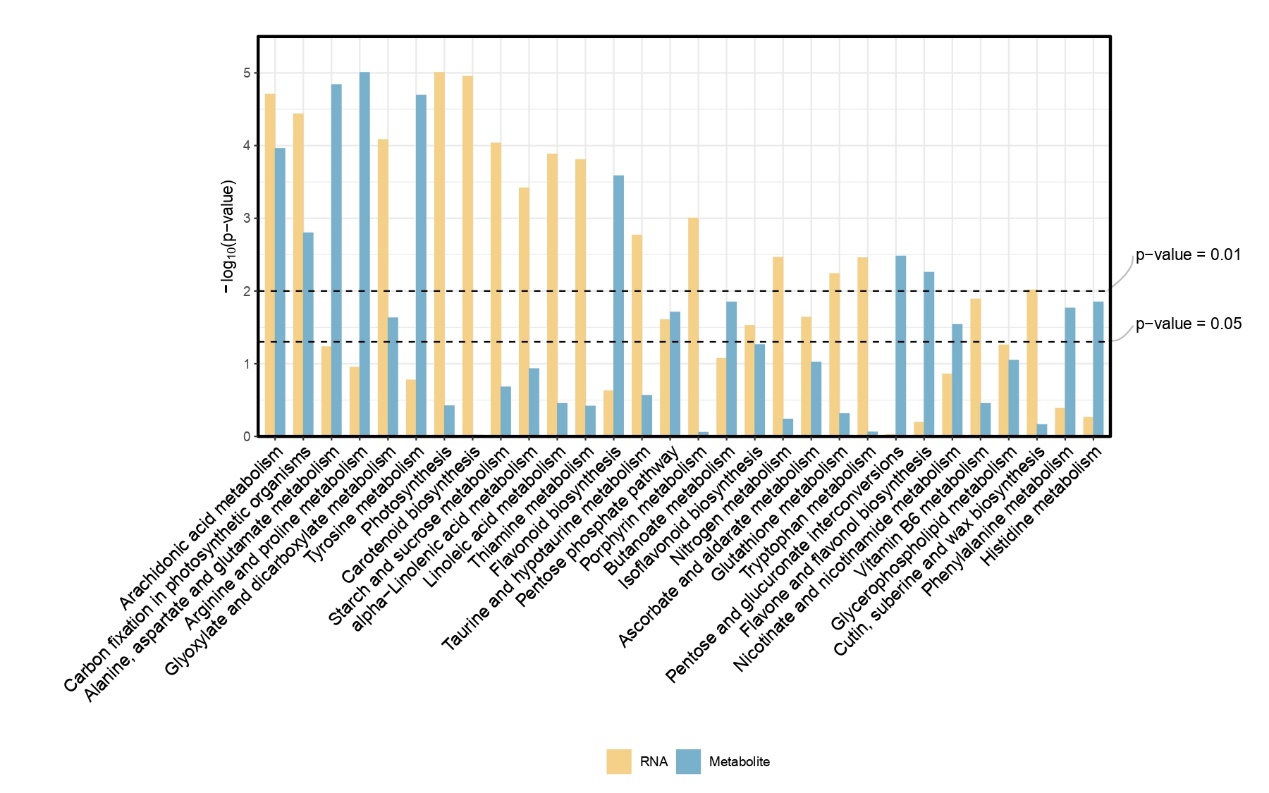

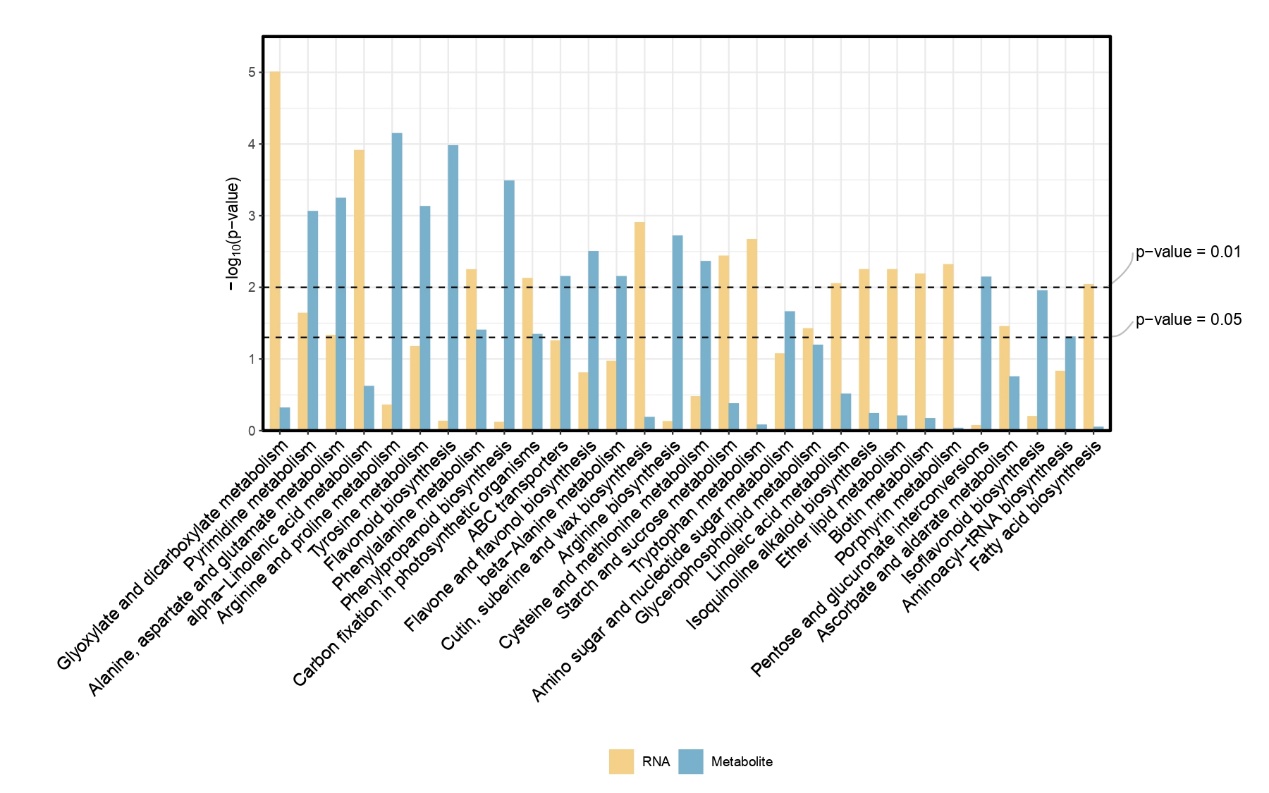


**B**

**A**

**SFig.6** KEGG Co-enrichment Plot of DEGs and DAMs, **A** Represents JG Material；**B** Represents TQ Material.


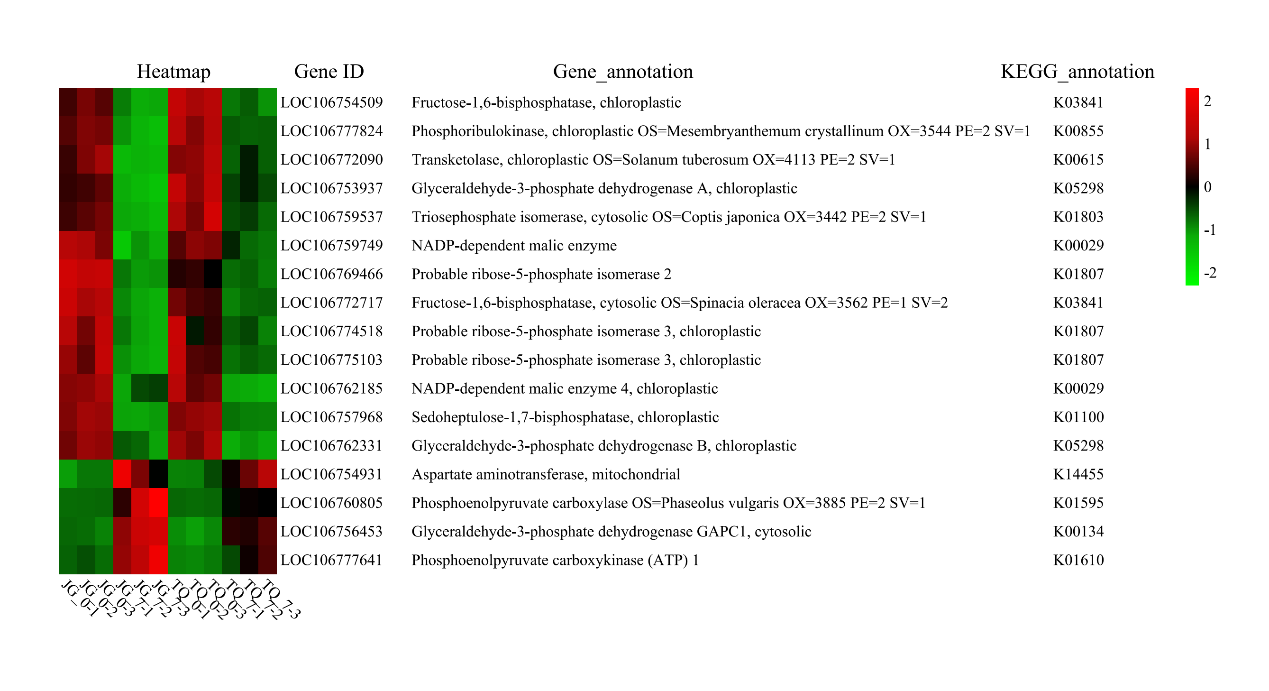


**SFig.7** Common Characteristics of JG and TQ Materials in Response to Saline-Alkali Stress in the Pathway of carbon fixation in photosynthetic organisms.


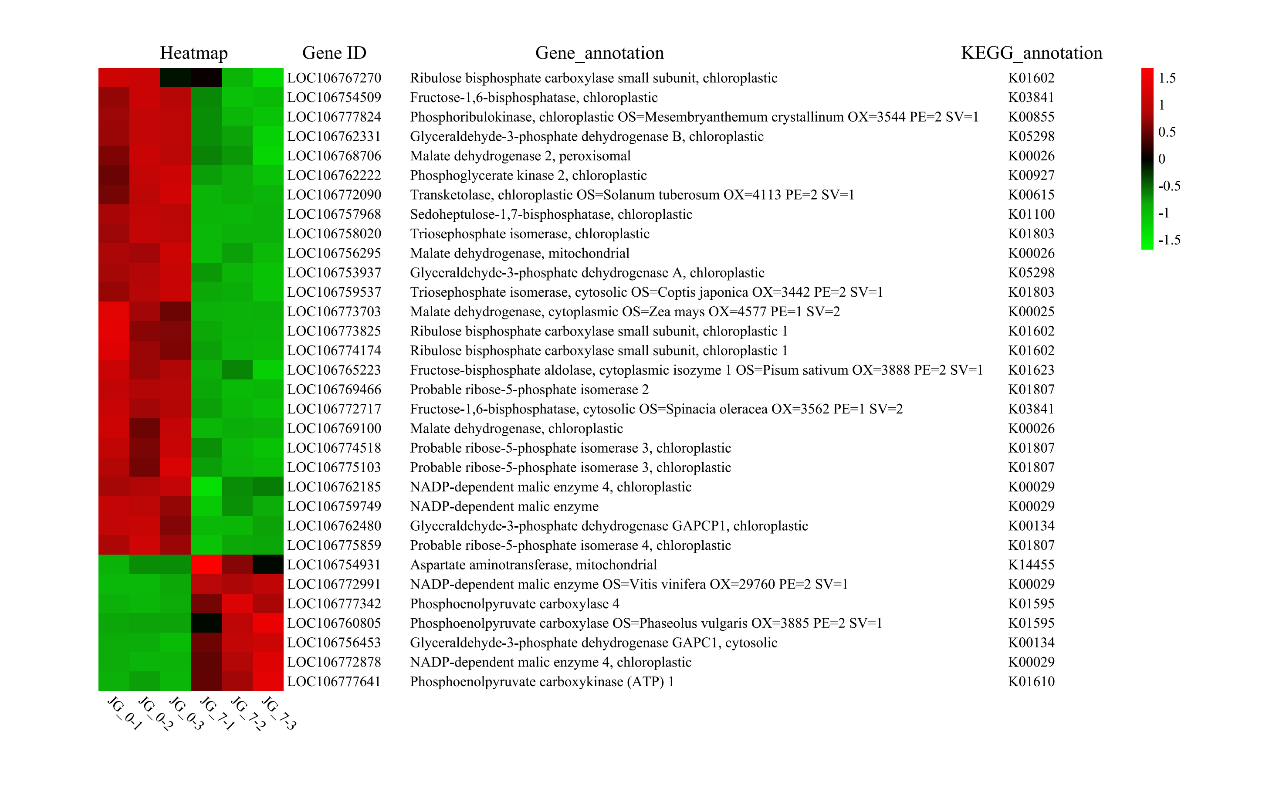


**A**


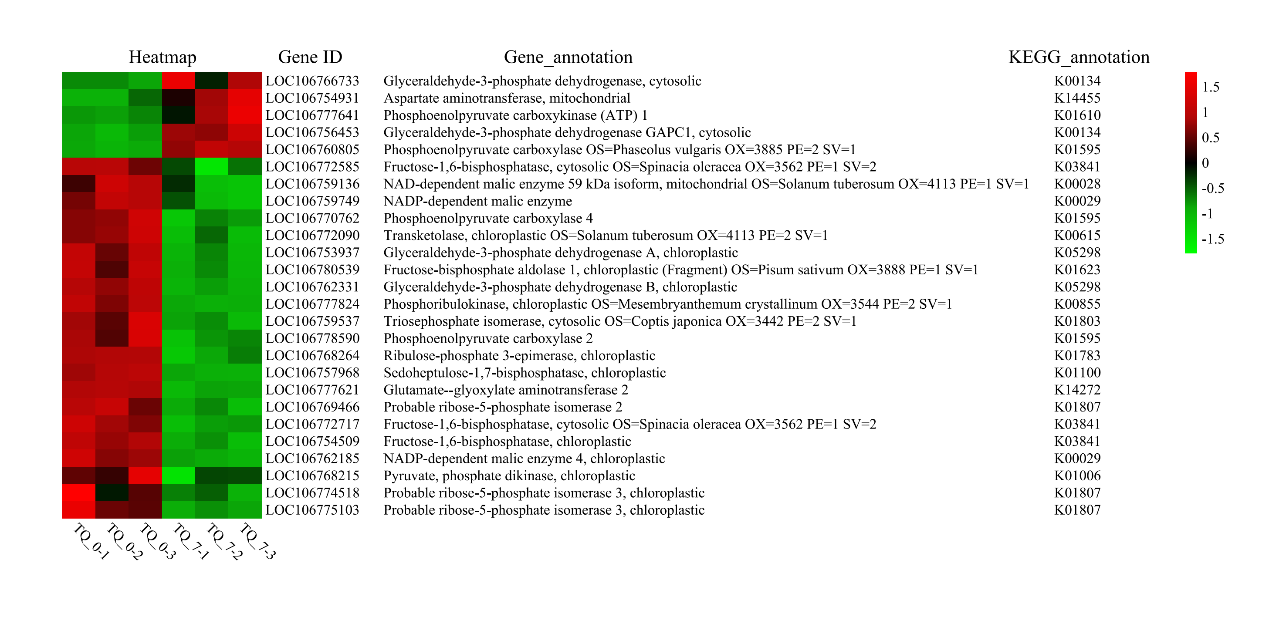
**SFig.8** **A** Characteristics of JG Material in Response to Saline-Alkali Stress in the Pathway of carbon fixation in photosynthetic organisms; **B** Characteristics of TQ Material in Response to Saline-Alkali Stress in the Pathway of carbon fixation in photosynthetic organisms

**B**


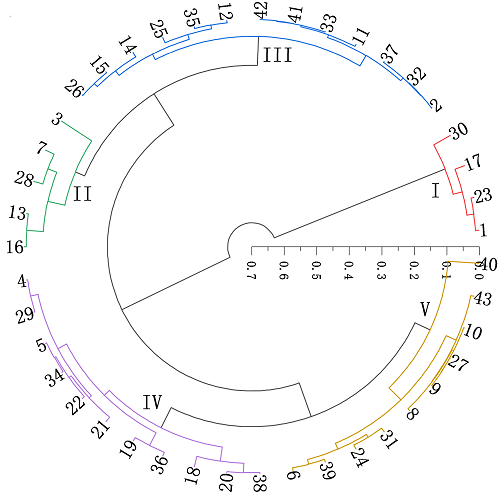


**SFig.9** Time series trend analysis of differential metabolites.

Note: No. 30 is JG material, No. 40 is TQ material.

Appendix Table 1 Index values of two materials before and after treatment

| Materials | Treatment | Indicator | | | | | |
| --- | --- | --- | --- | --- | --- | --- | --- |
|  |  | RL(cm) | GHAT(cm) | FW(g) | DW(g) | PWC | SPAD |
| JG | CK | 22.00±1.00a | 7.37±1.00a | 6.84±1.16a | 0.98±0.08a | 0.86±0.02a | 23.44±1.72a |
|  | 100mmol·L^-1^ | 21.40±2.80a | 5.80±0.80b | 5.95±1.00a | 0.95±0.23a | 0.84±0.01a | 21.46±0.90a |
| TQ | CK | 18.10±0.80a | 6.73±0.71a | 6.89±0.35a | 0.98±0.22a | 0.86±0.05a | 21.92±2.00a |
|  | 100mmol·L^-1^ | 14.30±2.30a | 1.65±0.41b | 3.48±0.40b | 0.59±0.01b | 0.83±0.00a | 18.96±0.59a |
|  |  | SS(mg/g) | MDA(nmol/g) | Pro(μg/g) | SOD(U/g) |  |  |
| JG | CK | 8.28±0.22a | 5.52±0.42a | 65.38±5.25a | 378.60±69.92a |  |  |
|  | 100mmol·L^-1^ | 6.92±1.12ab | 4.33±0.03b | 58.70±3.70b | 417.92±36.45a |  |  |
| TQ | CK | 10.05±1.05a | 5.03±0.07a | 52.38±0.94a | 353.60±50.88a |  |  |
|  | 100mmol·L^-1^ | 4.56±0.46b | 2.69±0.15b | 25.33±2.27b | 402.86±22.99a |  |  |
|  |  | Pn(umol·m-2·s-1) | Tr(mmol·m-2·s-1) | Gs(mmol·m-2) | Ci(ppm) |  |  |
| JG | CK | 3.79±0.25b | 20.35±2.00a | 44.07±0.02b | 399.63±50.68b |  |  |
|  | 100mmol·L^-1^ | 3.91±0.02b | 22.26±2.08a | 51.96±1.76b | 447.14±25.46ab |  |  |
| TQ | CK | 2.93±0.21b | 15.67±1.05b | 38.53±5.57c | 483.34±10.14c |  |  |
|  | 100mmol·L^-1^ | 3.49±0.25b | 21.00±4.50b | 65.40±5.42b | 640.48±40.33b |  |  |

Note: Different letters in the table indicate significant differences (*P* < 0.05).

Appendix Table 2 Comprehensive salt-alkali tolerance index values, weights, U(X_j_), D values and rankings of the test material

| Code | Cl_1_ | Cl_2_ | Cl_3_ | Cl_4_ | Cl_5_ | Cl_6_ | U(X_1_) | U(X_2_) | U(X_3_) | U(X_4_) | U(X_5_) | U(X_6_) | D value | Rank |
| --- | --- | --- | --- | --- | --- | --- | --- | --- | --- | --- | --- | --- | --- | --- |
| 1 | 2.580 | -0.250 | 0.318 | 0.626 | 0.576 | -0.248 | 0.994 | 0.580 | 0.551 | 0.705 | 0.613 | 0.542 | 0.700 | 3 |
| 2 | -1.140 | 0.602 | 1.031 | 0.590 | 1.470 | 1.299 | 0.122 | 0.748 | 0.737 | 0.698 | 0.815 | 0.826 | 0.466 | 17 |
| 3 | 0.185 | 0.227 | 1.365 | 0.533 | 1.975 | 0.423 | 0.432 | 0.674 | 0.824 | 0.686 | 0.929 | 0.665 | 0.576 | 5 |
| 4 | -0.748 | -0.403 | 0.164 | 0.479 | -0.460 | 1.681 | 0.213 | 0.550 | 0.510 | 0.676 | 0.380 | 0.896 | 0.403 | 29 |
| 5 | -1.464 | 0.560 | 1.475 | -0.521 | 0.981 | 0.325 | 0.045 | 0.740 | 0.852 | 0.476 | 0.705 | 0.647 | 0.410 | 28 |
| 6 | -0.332 | -0.302 | -0.123 | -1.247 | -1.408 | -0.255 | 0.311 | 0.569 | 0.436 | 0.331 | 0.166 | 0.541 | 0.360 | 35 |
| 7 | -0.209 | 0.482 | 0.883 | 0.843 | -0.048 | 0.562 | 0.340 | 0.724 | 0.698 | 0.748 | 0.473 | 0.690 | 0.507 | 9 |
| 8 | -0.579 | -0.449 | -1.794 | 0.223 | 0.787 | -0.203 | 0.253 | 0.540 | 0.000 | 0.624 | 0.661 | 0.550 | 0.341 | 38 |
| 9 | -0.788 | -1.676 | -0.125 | -0.361 | 2.291 | -1.161 | 0.204 | 0.298 | 0.435 | 0.508 | 1.000 | 0.374 | 0.338 | 40 |
| 10 | -0.451 | -3.186 | 0.336 | 0.827 | -0.077 | 1.127 | 0.283 | 0.000 | 0.555 | 0.745 | 0.466 | 0.794 | 0.339 | 39 |
| 11 | -0.314 | 1.468 | -0.268 | -1.430 | -0.552 | 1.661 | 0.315 | 0.919 | 0.398 | 0.294 | 0.359 | 0.892 | 0.458 | 22 |
| 12 | -0.523 | 1.878 | -0.103 | -0.725 | 0.941 | 0.150 | 0.266 | 1.000 | 0.441 | 0.435 | 0.695 | 0.615 | 0.480 | 14 |
| 13 | 0.398 | 0.536 | 0.685 | 0.571 | -0.301 | 0.143 | 0.482 | 0.735 | 0.646 | 0.694 | 0.416 | 0.614 | 0.541 | 6 |
| 14 | -0.147 | 1.454 | 0.400 | -1.769 | 0.218 | 0.629 | 0.355 | 0.916 | 0.572 | 0.226 | 0.533 | 0.703 | 0.488 | 12 |
| 15 | 0.243 | 1.483 | -0.975 | -0.544 | -0.449 | 1.205 | 0.446 | 0.922 | 0.214 | 0.471 | 0.382 | 0.809 | 0.495 | 11 |
| 16 | 1.038 | -0.159 | -0.530 | 0.696 | 0.004 | 0.286 | 0.633 | 0.598 | 0.330 | 0.719 | 0.484 | 0.640 | 0.538 | 7 |
| 17 | 2.552 | -0.150 | 0.869 | 0.131 | 0.041 | 0.492 | 0.988 | 0.599 | 0.694 | 0.606 | 0.493 | 0.678 | 0.711 | 2 |
| 18 | -1.533 | -0.051 | 0.878 | 0.260 | -0.652 | 2.247 | 0.029 | 0.619 | 0.697 | 0.632 | 0.336 | 1.000 | 0.373 | 33 |
| 19 | 0.636 | -1.071 | -1.774 | 0.222 | 0.841 | 0.368 | 0.538 | 0.418 | 0.005 | 0.624 | 0.673 | 0.655 | 0.433 | 24 |
| 20 | 0.189 | -1.296 | 1.498 | -2.900 | -1.582 | -0.655 | 0.433 | 0.373 | 0.858 | 0.000 | 0.127 | 0.467 | 0.386 | 32 |
| 21 | -0.694 | -0.356 | 0.102 | 1.117 | 0.886 | -0.086 | 0.226 | 0.559 | 0.494 | 0.803 | 0.683 | 0.571 | 0.420 | 25 |
| 22 | -0.204 | 0.094 | -0.800 | -0.527 | 0.973 | -0.391 | 0.341 | 0.648 | 0.259 | 0.475 | 0.703 | 0.515 | 0.415 | 26 |
| 23 | 2.504 | -0.530 | 1.094 | -0.139 | -0.007 | 0.529 | 0.977 | 0.525 | 0.753 | 0.552 | 0.482 | 0.684 | 0.695 | 4 |
| 24 | -0.401 | -1.128 | -1.148 | 0.206 | 0.282 | 0.098 | 0.295 | 0.406 | 0.169 | 0.621 | 0.547 | 0.605 | 0.349 | 37 |
| 25 | -0.150 | 0.426 | -0.443 | 1.154 | 0.547 | -0.034 | 0.354 | 0.713 | 0.352 | 0.811 | 0.607 | 0.581 | 0.473 | 15 |
| 26 | -0.266 | 1.036 | 0.177 | 0.697 | 0.068 | 0.421 | 0.327 | 0.834 | 0.514 | 0.719 | 0.499 | 0.665 | 0.496 | 10 |
| 27 | -0.590 | -1.508 | 0.075 | -0.077 | -0.213 | -0.633 | 0.250 | 0.331 | 0.487 | 0.564 | 0.435 | 0.471 | 0.338 | 41 |
| 28 | -0.261 | 1.610 | 1.027 | 0.407 | 0.017 | -1.184 | 0.328 | 0.947 | 0.736 | 0.661 | 0.487 | 0.370 | 0.520 | 8 |
| 29 | -0.078 | -0.227 | 0.527 | -0.839 | -2.144 | -0.346 | 0.371 | 0.584 | 0.605 | 0.412 | 0.000 | 0.524 | 0.402 | 30 |
| 30 | 2.604 | -0.208 | 1.153 | 0.078 | 0.312 | 0.606 | 1.000 | 0.588 | 0.768 | 0.595 | 0.554 | 0.699 | 0.729 | 1 |
| 31 | -0.200 | -0.492 | -1.523 | 0.084 | -0.447 | -0.473 | 0.342 | 0.532 | 0.071 | 0.597 | 0.383 | 0.500 | 0.355 | 36 |
| 32 | 0.159 | 0.865 | -0.836 | 0.125 | -0.350 | -0.139 | 0.426 | 0.800 | 0.250 | 0.605 | 0.404 | 0.562 | 0.467 | 16 |
| 33 | 0.193 | 0.727 | -1.165 | 0.364 | -0.324 | 0.112 | 0.434 | 0.773 | 0.164 | 0.653 | 0.410 | 0.608 | 0.461 | 19 |
| 34 | 0.178 | -0.499 | -1.602 | -0.231 | 0.731 | 0.568 | 0.431 | 0.531 | 0.050 | 0.534 | 0.648 | 0.692 | 0.412 | 27 |
| 35 | -0.336 | 0.877 | 0.485 | 2.102 | -0.832 | -1.409 | 0.310 | 0.802 | 0.594 | 1.000 | 0.296 | 0.329 | 0.482 | 13 |
| 36 | 0.168 | 1.193 | -1.531 | 1.148 | -2.118 | -0.611 | 0.428 | 0.865 | 0.069 | 0.809 | 0.006 | 0.475 | 0.438 | 23 |
| 37 | 0.633 | -0.230 | -0.862 | 0.426 | -0.980 | 0.177 | 0.537 | 0.584 | 0.243 | 0.665 | 0.262 | 0.620 | 0.465 | 18 |
| 38 | 0.108 | -0.266 | -1.181 | 0.054 | -1.019 | -0.747 | 0.414 | 0.577 | 0.160 | 0.591 | 0.254 | 0.450 | 0.388 | 31 |
| 39 | -0.180 | -0.125 | -0.629 | -1.386 | -0.400 | -1.295 | 0.347 | 0.604 | 0.304 | 0.303 | 0.393 | 0.349 | 0.364 | 34 |
| 40 | -1.093 | -0.684 | -0.487 | -1.773 | 0.539 | 0.792 | 0.133 | 0.494 | 0.341 | 0.225 | 0.605 | 0.733 | 0.304 | 43 |
| 41 | 0.196 | -0.114 | 1.099 | -1.594 | 0.510 | -1.932 | 0.435 | 0.607 | 0.754 | 0.261 | 0.598 | 0.232 | 0.460 | 20 |
| 42 | -0.228 | 0.900 | 0.216 | 0.107 | 1.345 | -3.198 | 0.336 | 0.807 | 0.524 | 0.601 | 0.787 | 0.000 | 0.460 | 21 |
| 43 | -1.658 | -1.053 | 2.041 | 1.990 | -1.973 | -0.901 | 0.000 | 0.421 | 1.000 | 0.978 | 0.038 | 0.422 | 0.335 | 42 |
| Weight | 0.3710 | 0.1994 | 0.1491 | 0.1080 | 0.0891 | 0.0834 |  |  |  |  |  |  |  |  |

Note: Cl_x_ represents the comprehensive index value derived from principal component analysis of the measured index values; U(_Xj_) stands for the membership function value; and the D value is the comprehensive evaluation value of salt‑alkali tolerance. No. 30 is JG material, No. 40 is TQ material.
